# Numerical cognition: A meta-analysis of neuroimaging, transcranial magnetic stimulation and brain-damaged patients studies

**DOI:** 10.1016/j.nicl.2019.102053

**Published:** 2019-10-22

**Authors:** Alexandrine Faye, Sophie Jacquin-Courtois, Emanuelle Reynaud, Mathieu Lesourd, Jérémy Besnard, François Osiurak

**Affiliations:** a Laboratoire d'Etude des Mécanismes Cognitifs (EA 3082), Université de Lyon, France; b Integrative, Multisensory, Perception, Action, & Cognition Team (INSERM-CNRS-UMR 5292), Université de Lyon, France; c Mouvement et Handicap, Hospices Civils de Lyon et Centre de Recherche en Neurosciences de Lyon, Hôpital Henry Gabrielle, St Genis Laval, France; d Aix Marseille Université, CNRS, LNC, Laboratoire de Neurosciences Cognitives, Marseille, France; e Aix Marseille Université, CNRS, Fédération 3C, Marseille, France; f Laboratoire de Psychologie des Pays de la Loire (EA 4638), Université de Nantes et d'Angers, France; g Institut Universitaire de France, Paris, France

**Keywords:** Triple code model, Symbolic format, Non-symbolic format, Numerical magnitude, Intraparietal sulcus, Angular Gyrus

## Abstract

This article offers the first comprehensive review examining the neurocognitive bases of numerical cognition from neuroimaging, Transcranial Magnetic Stimulation (TMS) and brain-damaged patients studies. We focused on the predictions derived from the Triple Code Model (TCM), particularly the assumption that the representation of numerical quantities rests on a single format-independent representation (i.e., the analogical code) involving both intraparietal sulci (IPS). To do so, we conducted a meta-analysis based on 28 neuroimaging, 12 TMS and 12 brain-damaged patients studies, including arithmetic and magnitude tasks in symbolic and non-symbolic formats. Our findings generally agree with the TCM predictions indicating that both IPS are engaged in all tasks. Nonetheless, the results of brain-damaged patients studies conflicted with neuroimaging and TMS studies, suggesting a right hemisphere lateralization for non-symbolic formats. Our findings also led us to discuss the involvement of brain regions other than IPS in the processing of the analogical code as well as the neural substrate of other codes underlying numerical cognition (i.e., the auditory-verbal code).

## Highlights

• We review neuroimaging, TMS, and patients studies on numerical cognition.
• We focused on the predictions derived from the Triple Code Model (TCM).
• Our findings generally agree with TCM predictions.
• Our results open avenues for the study of the neural bases of numerical cognition.

## 1 Introduction

Numbers are everywhere around us. We use them to know dates, to make a meal, to buy things. All cultures possess a minimum vocabulary to refer to quantities (Gordon, 2004; Pica et al., 2004). Several studies have also shown that all human beings can solve approximate and non-symbolic calculations (McCrink et al., 2013; Pica et al., 2004). Moreover, infants just a few months old are able to discriminate quantities (Izard et al., 2009; McCrink and Wynn, 2004; Wynn, 1992), corroborating that numerical skills are not restricted to our species. For instance, lionesses can assess the ratio of number of defenders to number of intruders before engaging in aggressive intergroup interactions (McComb et al., 1994; for a review, see Benson-Amram et al., 2017). Guppies can quantify very quickly and accurately small quantities (Agrillo et al., 2012; for a review see Agrillo and Bisazza, 2017), and young chicks can identify a target by its numerical serial position (Rugani et al., 2007; for a review see Rugani, 2017). Numerical skills can also be found in insects. For instance, honeybees can count the number of flower petals up to three (Gross et al., 2009; see also Dacke and Srinivasan, 2008; Skorupski et al., 2017). These observations are consistent with the theory of an innate number sense (Dehaene, 1997). According to this theory, even if the processing of symbolic numbers is proper to human beings – because of its language dependence –, many animals, including humans, possess an innate number sense, allowing them to represent and to compare quantities. In other words, this innate number sense is critical to process any quantity whatever the format (symbolic and non-symbolic). In humans, both intraparietal sulci (IPS; see Appendix for abbreviations of cerebral areas) might play a key role in the coding of this format-independent representation. This theory has become highly influential in the field of numerical cognition. Yet, no comprehensive review has been carried out to test its main predictions based on data from neuroimaging, TMS and brain-damaged patients. The aim of this article is to fill this gap.

### 1.1 The triple code model (TCM)

The number sense theory originates in the TCM introduced by Dehaene and colleagues (Dehaene, 1992; Dehaene and Cohen, 1995). In this model, three types of interconnected representations deal with the different numerical activities, namely, the *analogical magnitude representation*, the *verbal word frame* and the *visual Arabic form*.

The *analogic magnitude representation* (also called the analogical code) is a non-symbolic, preverbal number-representation, allowing to carry out comparison, estimation or approximate calculation tasks. In the TCM, this representation is central because it gives meaning to symbols: It contains the number sense. Because of it, we can perceive, understand and manipulate numerical quantities, whatever the format (symbolic or non-symbolic) or the modality. The number sense theory posits that this number sense is innate, automatic and common to all humans and some animals (Dehaene, 1997). It is the basis for two basic cognitive mechanisms. The first one is the subitizing, which provides direct perception of small quantities (up to 3 or 4) without counting (Kaufman et al., 1949; Mandler and Shebo, 1982; Spelke, 2008; Spelke and Kinzler, 2007; Trick and Pylyshyn, 1994; Xu et al., 2005). The second is the approximate number system, which is involved in larger quantities (beyond 4; Dehaene, 1997; Piazza et al., 2004; Piazza, 2010; Pica et al., 2004; Verguts and Fias, 2004). This system plays a crucial role in the representation of numerical quantity, also called mental number line, which resembles a compressed logarithmic curve. The corollary is that the degree of imprecision increases as the quantity to be estimated increases, according to a linear function: Large quantities are less well represented than small quantities (Halberda et al., 2008). Moreover, it is easier to determine which quantity is the greater when the two quantities are distant than when they are close (e.g., it is easier to compare 28–95 than 28–32). This effect, called the distance effect, is also observed in non-symbolic formats. Finally, the size effect reveals that, for a given distance, comparison difficulty increases with increasing size (e.g., it is easier to compare 4–5 than 18–19; Dehaene et al., 1998).

The two other representations are symbolic and culture-dependent. *The verbal word frame* (also called the auditory-verbal code) represents numbers as organized sequences of words. It is the basis for counting skills as well as the retrieval of arithmetic facts stored in long-term memory (Dehaene and Cohen, 1995). Arithmetic facts refer to one-digit operations memorized by rote learning, for which a solution is stored in relation to an arithmetic problem (De Smedt, 2018). In the *visual Arabic form* (also called the Arabic code), quantities are represented as a sequence of numbers. It is the visual form of numbers. This representation is critical to recognize and write Arabic digits as well as to carry out multi-digit operations and to perform parity decisions (Dehaene and Cohen, 1995).

**Fig. 1:**
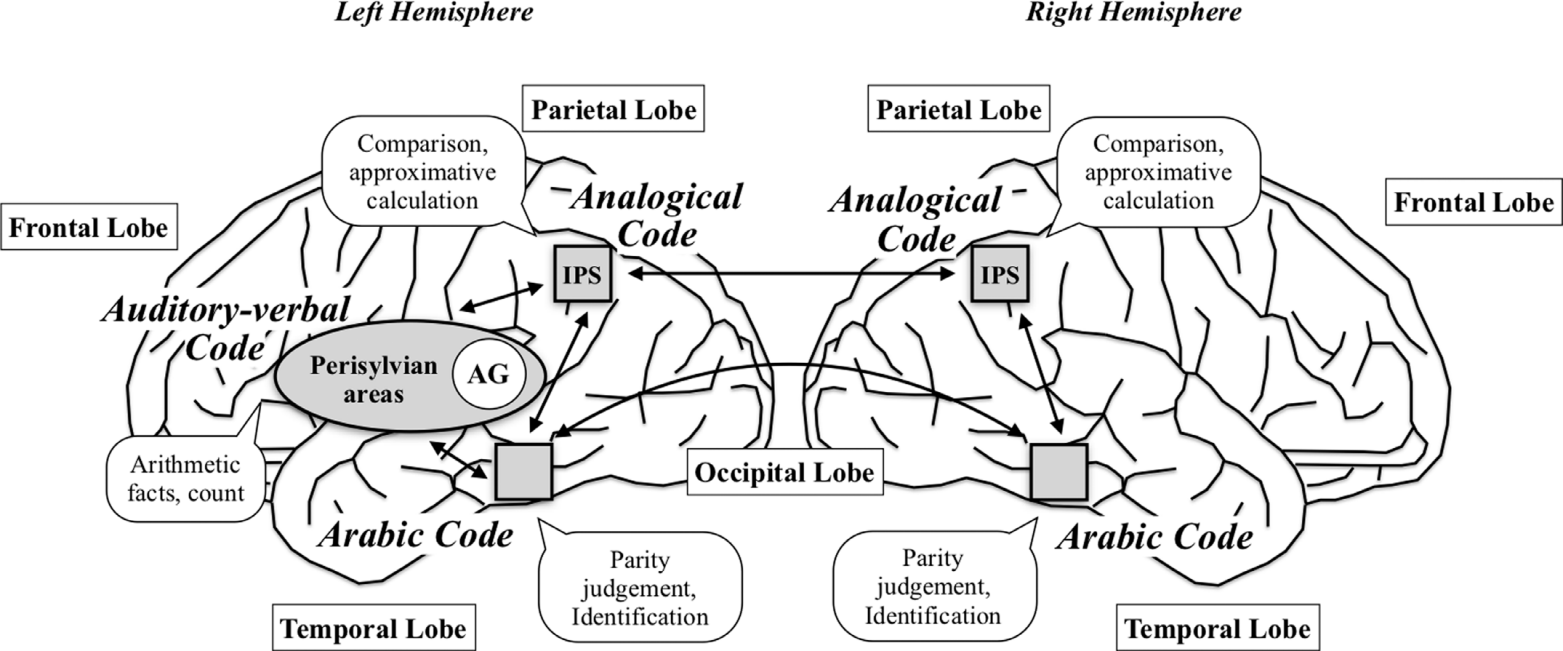
*The Triple Code Model.* This figure, based on Dehaene and Cohen (1995), represents an external view of the neuroanatomical implementation of the TCM (Dehaene and Cohen, 1995). The arrows illustrate the diffusion of information between the different codes and from one hemisphere to another, rather than the real neural fiber network.

Dehaene and Cohen (1995) proposed a neuroanatomical implementation of the TCM (Fig. 1). More particularly, they hypothesized that both IPS possess an analogical representation of numerical quantities (i.e., the analogical code). Therefore, these cerebral areas might be recruited for all numerical tasks (calculation, comparison, etc.) as soon as the task requires access to a representation of numerical quantity (Dehaene et al., 2003). In addition, both IPS might be involved in numerical skills whatever the format or the modality. Besides, they suggested that the left angular gyrus (AG) and perisylvian areas might play a key role when arithmetic operations impose strong requirements in terms of verbal coding of numbers (retrieval of arithmetic facts; i.e., the auditory-verbal code; see Dehaene et al., 2003; see also Dehaene et al., 1999; Delazer et al., 2003; Grabner et al., 2009; Venkatraman et al., 2006). Finally, the left and right occipito-temporal regions (i.e., the ventral visual stream) might be critical for the Arabic code (Dehaene and Cohen, 1995; Piazza and Eger, 2016; Shum et al., 2013).

### 1.2 Experimental evidence for the TCM

#### 1.2.1 Neuroimaging studies

The TCM predicts that both IPS possess format-independent analogical representations (e.g., Dehaene and Cohen, 1995; Dehaene et al., 2003). Many neuroimaging studies have confirmed this prediction, showing bilateral activation of the IPS for both formats, i.e., in symbolic (Ansari et al., 2006, 2005; Chochon et al., 1999; Lyons et al., 2015; Piazza et al., 2007; Venkatraman et al., 2005) and non-symbolic tasks (Cantlon et al., 2006; Lyons et al., 2015; Piazza et al., 2007; Venkatraman et al., 2005). Furthermore, evidence has indicated bilateral activation of the IPS for magnitude tasks (e.g., comparison) with Arabic digits (Piazza et al., 2007), number-words (Pinel et al., 2001) or non-symbolic formats (Dormal and Pesenti, 2009; Piazza et al., 2007). This activation has been found for calculation tasks (Chochon et al., 1999), including addition (Stanescu-Cosson et al., 2000; van der Ven et al., 2016) and subtraction tasks (Simon et al., 2002). Besides, Delazer et al. (2003) found that the IPS showed significant activations during the training period of new arithmetic facts (i.e., multiplication). Nonetheless, once the arithmetic fact was learned, there was a shift of activation from the IPS to the left AG, suggesting a modification from quantity-based processing to an automatic retrieval of learned arithmetic facts.

#### 1.2.2 TMS and brain-damaged patients studies

Transcranial magnetic stimulation (i.e., TMS) studies have also provided evidence for the TCM. The stimulation of the IPS deteriorates the performance in magnitude tasks (e.g., numerical order judgment or comparison) in non-symbolic (Lecce et al., 2015) or symbolic formats (Andres et al., 2005; for addition symbolic tasks, see Salillas et al., 2012). It has also been shown that a virtual lesion of the portion of the posterior IPS reduced the SNARC effect^1^ (i.e., the Spatial Numerical Association of Response Code; Dehaene et al., 1993) in a parity judgment task, confirming the involvement of the IPS in the mental number line (Rusconi et al., 2007). The potential role of the left AG for the auditory-verbal code has also been corroborated by TMS studies. For instance, Maurer et al. (2015) found that stimulation of the left AG disrupted the performance in different simple calculation tasks.

Studies in brain-damaged patients have also strengthened the format-independent hypothesis of IPS, even though theses studies have focused more on the dissociation between magnitude and arithmetic operations rather than between symbolic versus non-symbolic formats (Ashkenazi et al., 2008; Cohen and Dehaene, 1996; Cohen et al., 2000; Lemer et al., 2003; Sandrini et al., 2003). For instance, Ashkenazi et al. (2008) presented a single-case patient, AD, with left IPS lesion. AD had difficulties to perceive and manipulate symbolic and non-symbolic quantities and was impaired for complex operations. However, AD had no major difficulties in solving arithmetic facts. The case study of a female, split-brain patient of Cohen and Dehaene (1996) revealed that each hemisphere was able to identify Arabic digits and to manipulate discrete quantities (i.e., the analogical code). Interestingly, the transfer from the right hemisphere to the left hemisphere was impossible for Arabic digits, but possible in non-symbolic formats. Moreover, only the left hemisphere could perform arithmetic operations. This case study confirmed that both hemispheres support the analogical code, whereas only the left hemisphere possesses a complete calculation system (i.e., the auditory-verbal code). Several group studies in right brain-damaged (RBD) patients have also indicated greater impairment in numerical magnitude processing than calculation (Dellatolas et al., 2001; Rosselli and Ardilla, 1989; Warrington and James, 1967). For instance, Masson et al. (2013) showed that RBD patients with neglect were impaired in symbolic and non-symbolic numerical order judgment tasks with small quantities, suggesting that neglect prevents from orienting attention to the left of the mental number line (see also Masson et al., 2015).

### 1.3 Overview

The goal of this article was to test the main predictions derived from the TCM (for a summary, see Table 1). We notably focused on the assumption that the representation of numerical quantities rests on a single format-independent representation (i.e., the analogical code) involving both IPS. We were also interested in the link between arithmetic facts and left AG. To achieve our goal, we conducted a meta-analysis based on 28 neuroimaging, 12 TMS and 12 brain-damaged patients studies, including arithmetic and magnitude tasks (hereafter referred to as ARITHMETIC and MAGNITUDE) in symbolic and non-symbolic formats (hereafter referred to as SYMBOLIC and NON-SYMBOLIC).

**Table 1:** Predictions derived from the Triple Code Model (TCM).

Quantities (Identification, Magnitude)	Non Symbolic		Intraparietal Sulci (IPS)
	Symbolic	Arabic digits	Intraparietal Sulci (IPS)
		Number-word	Intraparietal Sulci (IPS)
Exact calculation (One-digit operation)	Symbolic	Addition	Intraparietal Sulci (IPS)
			Left Angular Gyrus (AG)
			Left Perisylvian Areas
		Subtraction	Intraparietal Sulci (IPS)
		Multiplication	Left Angular Gyrus (AG)
			Left Perisylvian Areas
		Division	Left Angular Gyrus (AG)
			Left Perisylvian Areas
Approximate calculation	Non Symbolic		Intraparietal Sulci (IPS)
	Symbolic		Intraparietal Sulci (IPS)

## 2 Methods

We examined the key brain areas associated to (1) SYMBOLIC and NON-SYMBOLIC and (2) ARITHMETIC and MAGNITUDE. Nevertheless, some overlapping could occur between, for instance, NON-SYMBOLIC and MAGNITUDE. More specifically, it is noteworthy that NON-SYMBOLIC included only magnitude tasks, whereas SYMBOLIC could include both arithmetic and magnitude tasks. Conversely, ARITHMETIC included only tasks in symbolic formats (except for one fMRI study using a non-symbolic addition task), whereas MAGNITUDE included tasks in both non-symbolic and symbolic formats.

### 2.1 Selection of studies

We identified relevant studies for inclusion using two databases: PubMed and PsychInfo. We limited our search to studies published between October 1967 and March 2017. We selected relevant studies based on several selection criteria such as: Neuroimaging (fMRI or PET; neurologically healthy adults; Whole brain analysis), TMS (repetitive Transcranial Magnetic Stimulation or continuous Theta Burst Stimulation; neurologically healthy adults; Right or left hemisphere), and brain-damaged patients (Unilateral lesions, stroke or tumor; Group studies; Presence of a control group). More detail about the selection of studies is provided in **Supplementary Material**.

### 2.2 Data analysis

#### 2.2.1 Neuroimaging

Our meta-analysis was performed with the revised version (Eickhoff et al., 2009, 2012) of the activation likelihood estimation method (ALE; Turkeltaub et al., 2002), as applied by the GingerALE 2.3 software (http://www.brainmap.org/ale/). ALE is a coordinate-based method for grouping neuroimaging studies findings. The aim of the ALE method is to identify brain regions that are reliably activated throughout studies. Based on the stereotactic coordinates of activation peaks gathered in each study involved in the meta-analysis, this method assesses at each voxel the probability that an activation focus actually exists within that given voxel, under Gaussian assumptions on spatial uncertainty. The unification of the voxel-wise of probabilities over all activation foci allows to create an ALE map. Clusters of significantly high ALE are the significantly overlapping clusters of activation, discovering a convergence throughout included imaging studies.

To carry out this meta-analysis, coordinates of each significant activation peak for all conditions included were considered. The meta-analysis was conducted in the Talairach reference space (Talairach and Tournoux, 1988). Coordinates that were presented in the Montreal Neurological Institute (MNI) space were first converted to Talairach space using the icbm2tal transformation (Lancaster et al., 2007) applied in the GingerALE software. For every study included and at every voxel, ALE computes the likelihood that an activation focus is located at this voxel location. To consider the spatial uncertainty, foci are regarded as to be the centers of three-dimensional Gaussian likelihood density functions. Full widths at half maximum of 3D Gaussian functions (FWHM) require the sample size: Studies with a larger sample size therefore had a stronger impact on the results.

The likelihood distributions of all foci in the investigated experiment are united in a Modeled Activation (MA) map. The union of all MA maps for all the experiments involved in the meta-analysis permits computing an ALE score on a voxel-by-voxel basis. This score quantifies the probability of concurrent activations at each voxel throughout all included studies. Significance tests are performed by comparing the ALE scores with a null distribution acquired from the same number of randomly placed activation foci. At the condition level, all foci from a generic contrast are brought together: The ensuing *p*-values are then limited at a false discovery rate (FDR) of *p* < 0.05, and only clusters of a minimum volume of 100 mm^3^ are presented. For particular contrasts between two conditions (subtraction analysis), ALE individual maps related to each condition were limited at a level of *p* < 0.05 (FDR corrected) as was the pooled map for the two conditions. The contrast analysis was then carried out on these maps and the results were mentioned with a *p*-value threshold set to *p* < 0.05 and minimum cluster sizes set to 100 mm^3^ (Laird et al., 2005; Turkeltaub et al., 2012). The ensuing thresholded ALE maps were depicted on flat-map representations of a standardized brain atlas (PALS-B12: Population-Average, Surface- and Landmark-based human cortical atlas; Van Essen, 2005), using Caret, version 5.65 (http://brainmap.wustl.edu/caret.html; Van Essen et al., 2001).

#### 2.2.2 TMS

The virtual lesions were processed from the information obtainable (e.g., stimulation coordinates) in the studies involved in this meta-analysis. First, we transformed the stimulation coordinates that were presented in MNI space into Talairach-coordinates (Lacadie et al., 2008). Second, every coordinate was illustrated on a flat-map representation of the corresponding hemisphere (PALS-B12: Population-Average, Surface- and Landmark-based human cortical atlas; Van Essen, 2005), using Caret, version 5.65 (http://brainmap.wustl.edu/caret.html; Van Essen et al., 2001). Third, the coordinate was depicted by a specific symbol depending on the stimulation effect (i.e., Star: Deficit; Sphere: Normal), a color according to the nature of format (i.e., Purple: SYMBOLIC; Green: NON-SYMBOLIC) and the type of task (i.e., Red: ARITHMETIC; Blue: MAGNITUDE), and a number corresponding to each illustrated stimulation (e.g., 1: Göbel et al., 2001a).

#### 2.2.3 Brain-damaged patients

We could not perform a quantitative meta-analysis because effect sizes and standard errors were not always available. We conducted a qualitative meta-analysis, focusing on the mean raw scores (i.e., correct responses) obtained by the patient group based on the information available in the studies included in this meta-analysis. First, we categorized the tasks of the studies according to the format (i.e., SYMBOLIC versus NON-SYMBOLIC) and the type (i.e., ARITHMETIC versus MAGNITUDE). Second, for each task, mean raw scores obtained by the patient group were converted to percents by dividing each raw score by the maximum score on the task (e.g., the mean raw score obtained by a patient group is 48, the maximum score is 60, so 48/60×100 = 80%). We followed the same procedure for matched control groups. Third, we calculated a point-score for each patient group, corresponding to the difference between the percent score of the patient group and that of the matched control group (e.g., a percent score of 80% for the patient group minus a percent score of 96% for the control group = *a* point-score of −16%; see Lesourd et al., 2013 for a similar procedure). The lower the point-score, the more the patient group is impaired compared to the control group. Fourth, we used a graphical illustration of the results obtained for LBD and RBD patients separately, in representing each patient group by a rectangle on a vertical axis. The size of the rectangle depended on the number of participants (i.e., the height; vertical axis) and the point-score (i.e., the length; horizontal axis). The different patient groups were positioned in ascending order: From the negative point-scores (i.e., patient group < control group) to the positive point scores (i.e., patient group > control group). Moreover, each color of rectangles corresponds to a study (e.g., Purple: Dellatolas et al., 2001) and the stars alongside the rectangles indicate a significant difference between the performance obtained by the patient group and the control group (information based on the studies). Note that in order to facilitate the comparison between LBD and RBD patients, the vertical axis had to be of the same height. Therefore, the unit used for the vertical axis could vary according to the total number of patients for a specific condition. Fifth, for a specific condition (e.g., SYMBOLIC), we superposed in grey the area covered by the point-scores of LBD patients on the point scores of RBD patients, and vice versa, in order to facilitate the comparison of performance between LBD and RBD patients.

## 3 Results

### 3.1 Neuroimaging

Our goal was to test the predictions derived from the TCM. So, we will focus here mainly on the two main regions of interest (IPS and AG) and will detail to a lesser extent cerebral areas (e.g., frontal) that are secondary for numerical cognition in accordance with the TCM. Note that the key findings of the meta-analyses conducted here from neuroimaging studies in the different conditions (SYMBOLIC and NON-SYMBOLIC; ARITHMETIC and MAGNITUDE; SYMBOLIC MAGNITUDE and NON-SYMBOLIC MAGNITUDE) are shown in Table 2.

**Table 2:** Key findings from neuroimaging.

Condition	Symbolic		Non-Symbolic		Arithmetic		Magnitude		Symbolic Magnitude		Non-Symbolic Magnitude	
Hemisphere	Left	Right	Left	Right	Left	Right	Left	Right	Left	Right	Left	Right
Main regions of interest												
IPS	*	*	*	*	*	*	*	*	*	*	*	*
AG	*		*		*		*		*			
Secondary areas												
SMG	*	*		*	*	*	*	*	*			*
IFC	*	*	*	*	*	*	*		*		*	*
IFG	*		*	*	*		*	*	*			*
DLPFC	*	*		*	*	*						*
MTG	*				*							
SFL	*				*							

IPS, Intraparietal Sulcus; AG, Angular Gyrus; SMG, Supramarginal Gyrus; IFC, Inferior Frontal Cortex; IFG, Inferior Frontal gyrus; DLPFC, Dorso-Lateral Prefrontal Cortex; MTG, Middle Temporal Gyrus; SFL, Superior Frontal Language area.3.1.1. Overview.

**Fig. 2:**
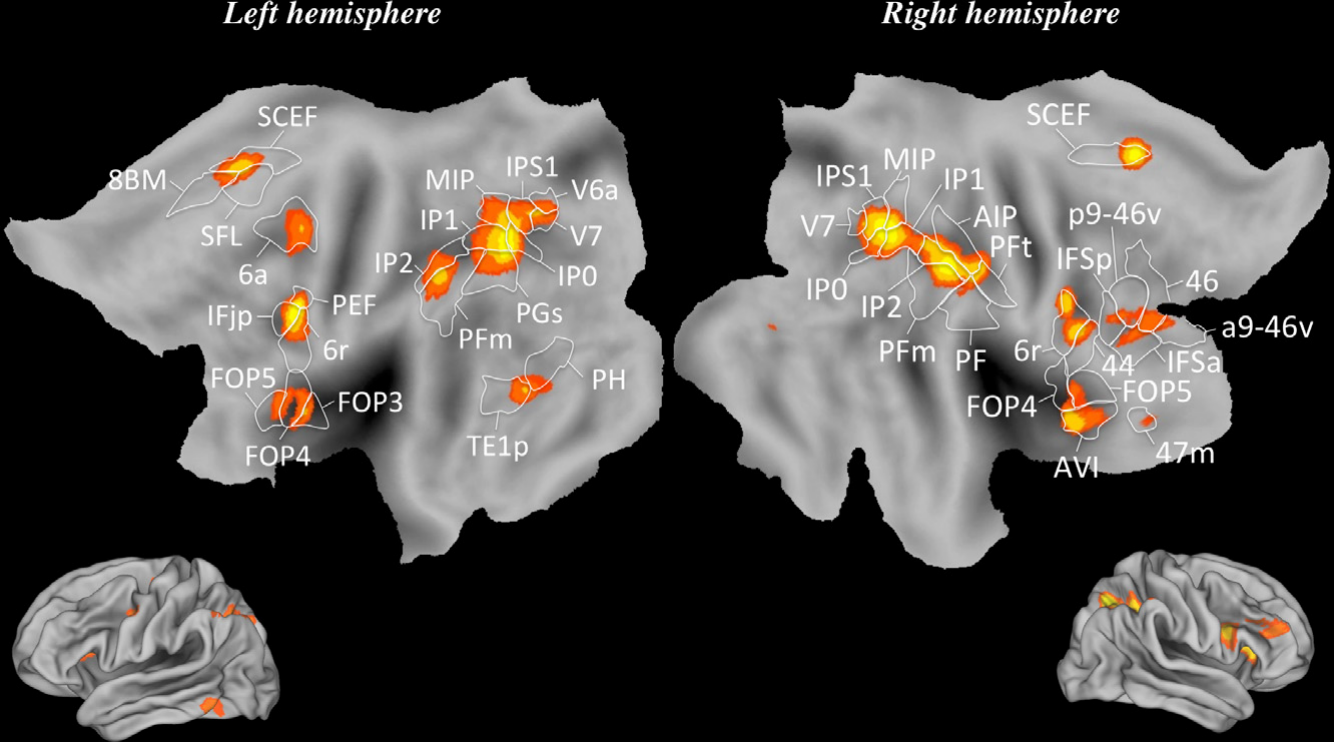
The “numerical cognition circuit”. ALE map derived from all studies included, viewed on two PALS-B12 left and right atlas surface configurations (Van Essen, 2005). Flat maps (Top) and lateral fiducial maps (Bottom). The parcellation is based on Glasser et al. (2016). For abbreviations and explanation, see the main text and Appendix.

We highlighted a “numerical cognition circuit” corresponding to the overlap of regions of interest activated in all the included studies (Fig. 2). More specifically, the IPS was recruited bilaterally, at the level of the medial intraparietal area [MIP], the right anterior intraparietal area [AIP], the areas intraparietal 0, 1, 2 [IP0, IP1, IP2] and the intraparietal sulcus area 1 [IPS1]). The angular gyrus (left PGs) was also activated, but only in the left hemisphere. A set of secondary regions was recruited bilaterally: The supramarginal gyrus (PFm, right PFt, right PF), the inferior frontal cortex (left IFjp, right IFSp, right IFsa, right 44) and the inferior frontal gyrus (FOP5, left FOP3, left FOP4). In the left hemisphere, the DLPFC (46, p9-46v, a9-46v), the superior frontal language area (SFL) and the middle temporal gyrus (TE1p) were activated.

#### 3.1.2 Symbolic and non-symbolic

**Fig. 3:**
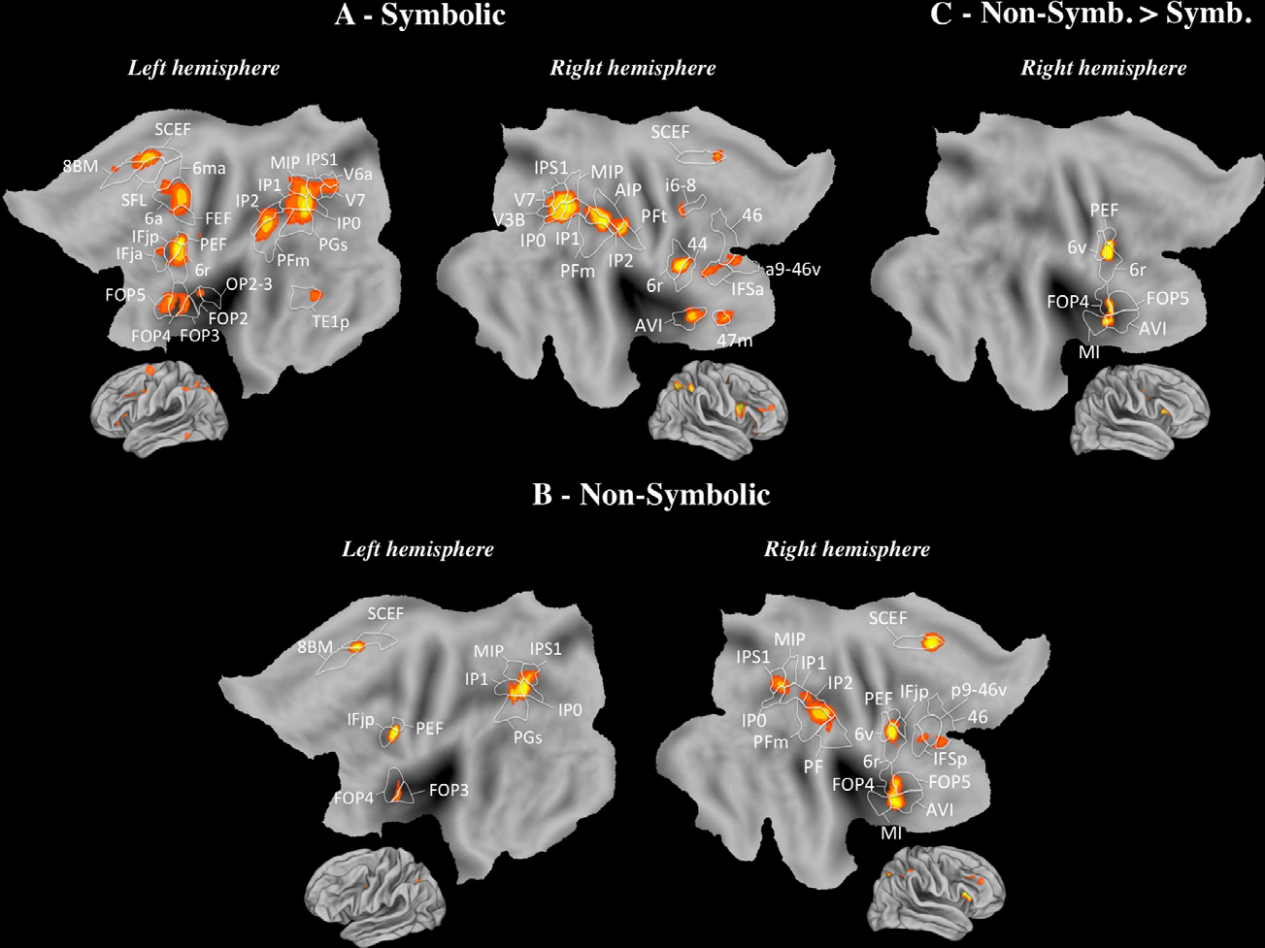
SYMBOLIC and NON-SYMBOLIC (Neuroimaging). ALE map derived from the studies included in (A) SYMBOLIC and (B) NON-SYMBOLIC, and (C) NON-SYMBOLIC < SYMBOLIC contrasts, viewed on PALS-B12 left and right atlas surface configurations (Van Essen, 2005). Flat maps (Top) and lateral fiducial maps (Bottom). The parcellation is based on Glasser et al. (2016). For abbreviations and explanation, see the main text and Appendix.

The results of the meta-analysis conducted separately for SYMBOLIC and NON-SYMBOLIC are displayed in Fig. 3A and Fig. 3B, respectively. For SYMBOLIC, we found bilateral activation in the IPS (MIP, IP0, IP1, IP2, IPS1, right AIP) and activation in the left AG (PGs). Secondary areas were also activated in the supramarginal gyrus (PFm, right PFt), the inferior frontal cortex (left IFjp and IFja, right IFsa and 44), the right DLPFC (right i6-8, 46, a9-46v), the left inferior frontal gyrus (FOP3, FOP4, FOP5, OP2-3), the SFL and the left middle temporal gyrus (TE1p). For NON-SYMBOLIC, bilateral activations occurred in the IPS (IP0, IP1, MIP, IPS1, right IP2) and the left AG (PGs). We found activation in secondary areas in the suparmarginal gyrus (right PFm, PF), the inferior frontal cortex (IFjp, right IFSp), the inferior frontal gyrus (FOP4, left FOP3, right FOP5), and the DLPFC (p9-46v, 46). Statistical comparisons were carried out to identify brain regions responding more reliably to SYMBOLIC than NON-SYMBOLIC and vice versa. The SYMBOLIC > NON-SYMBOLIC contrast did not reveal activation. The NON-SYMBOLIC > SYMBOLIC contrast indicates activation in secondary areas, and particularly, in the right inferior frontal gyrus (FOP4, FOP5; Fig. 3C).

#### 3.1.3 Arithmetic and magnitude

**Fig. 4:**
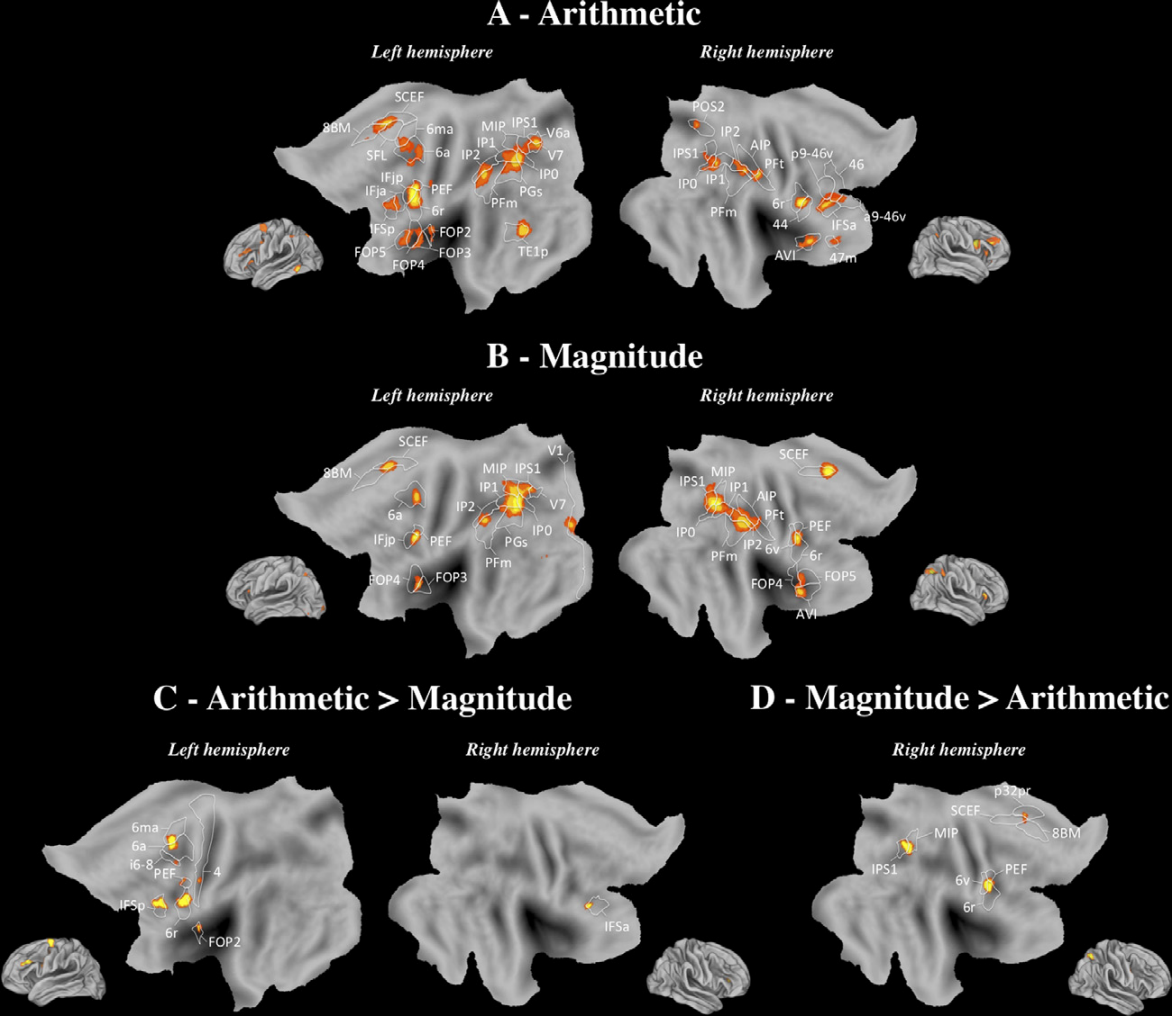
ARITHMETIC and MAGNITUDE (Neuroimaging). ALE map derived from the studies included in (A) ARITHMETIC and (B) MAGNITUDE, and (C) the ARITHMETIC > MAGNITUDE, and (D) MAGNITUDE > ARITHMETIC contrasts, viewed on PALS-B12 left and right atlas surface configurations (Van Essen, 2005). Flat maps (Top) and lateral fiducial maps (Bottom). The parcellation is based on Glasser et al. (2016). For abbreviations and explanation, see the main text and Appendix.

The results of the meta-analysis conducted separately for MAGNITUDE and ARITHMETIC are displayed in Fig. 4A and Fig. 4B, respectively. For ARITHMETIC, the IPS (IP0, IP1, IP2, IPS1, left MIP, right AIP) and the left AG (PGs) were activated. Concerning secondary areas, activation was found in the supramarginal gyrus (PFm, right PFt), the inferior frontal cortex (left IFSp, IFja and IFjp, right IFSa and 44), the left inferior frontal gyrus cortex (FOP2, FOP3, FOP4, FOP5), the left SFL, the left middle temporal gyrus (TE1p), and the right DLPFC (p9-46v, 46, a9-46v). MAGNITUDE activated the IPS (IP0, IP1, IP2, MIP, IPS1, right AIP) and the left AG (PGs). We also found activation in secondary areas and particularly in the supramarginal gyrus (PFm, right PFt), the left inferior frontal cortex (IFjp), and the inferior frontal gyrus (FOP4, left FOP3, right FOP5). Statistical comparisons were conducted to identify brain regions that were specifically activated for one condition compared to the other. The ARITHMETIC>MAGNITUDE contrast revealed activation in secondary areas, namely, the inferior frontal cortex (IFSp) and the inferior frontal gyrus (FOP2) (Fig. 4C). The right IPS (MIP, IPS1) was preferentially recruited for MAGNITUDE when contrasted to ARITHMETIC (Fig. 4D).

#### 3.1.4 Symbolic and non-symbolic magnitude

**Fig. 5:**
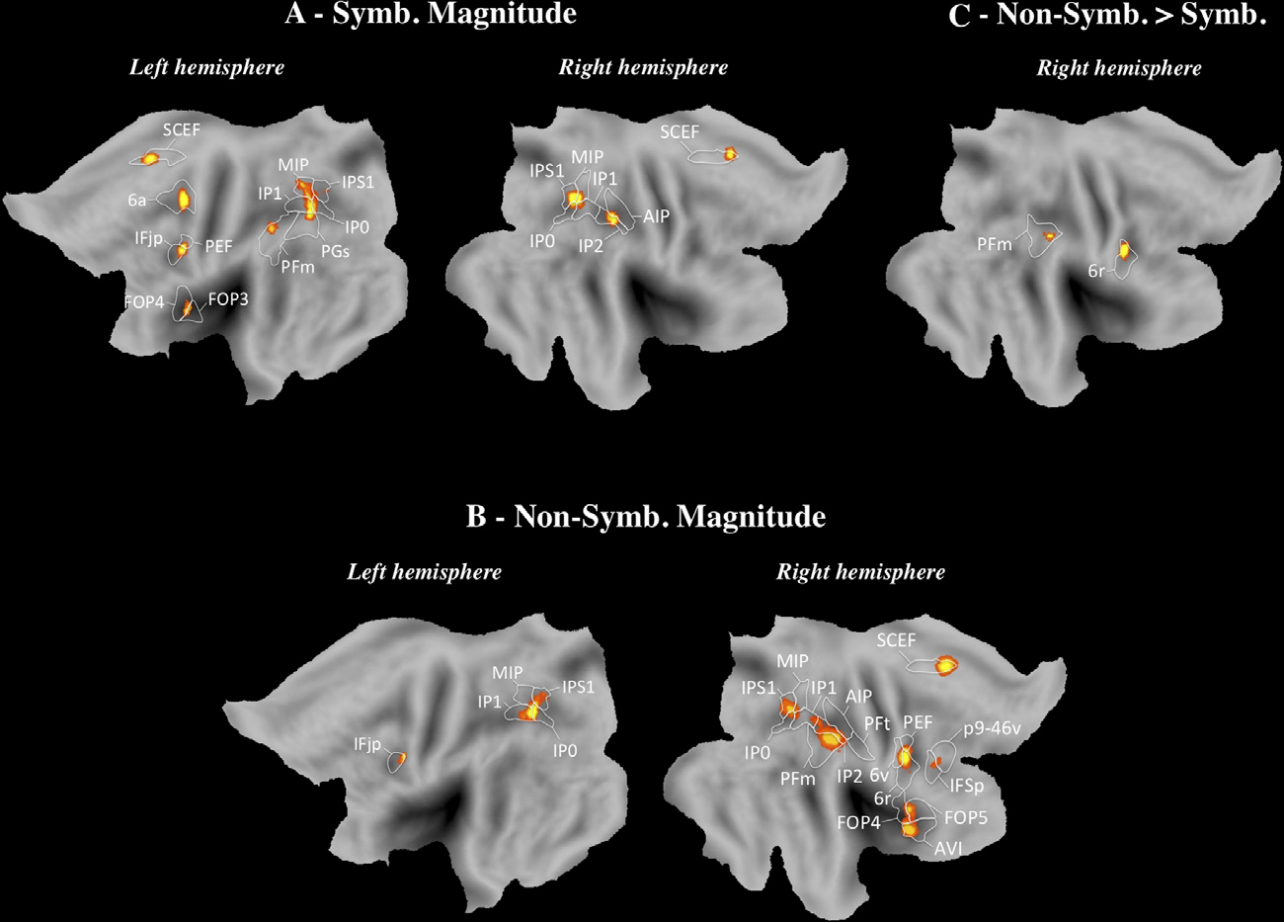
SYMBOLIC MAGNITUDE and NON-SYMBOLIC MAGNITUDE (neuroimaging). ALE map derived from the studies included in (A) SYMBOLIC MAGNITUDE and (B) NON-SYMBOLIC MAGNITUDE, and (C) the NON-SYMBOLIC MAGNITUDE > SYMBOLIC MAGNITUDE contrast, viewed on PALS-B12 left and right atlas surface configurations (Van Essen, 2005). The parcellation is based on Glasser et al. (2016). For abbreviations and explanation, see the main text and Appendix.

As mentioned above, the interpretation of results obtained for MAGNITUDE is delicate given that MAGNITUDE included tasks in symbolic and non-symbolic formats. So, we conducted an additional analysis by distinguishing between SYMBOLIC MAGNITUDE (Fig. 5A) and NON-SYMBOLIC MAGNITUDE (Fig. 5B). For SYMBOLIC MAGNITUDE, significant activations were found in the IPS (IP0, IP1, IPS1, MIP, right IP2 and AIP) and the left AG (PGs). Activation in secondary areas also occurred, particularly in the supramarginal gyrus (PFm), the inferior frontal cortex (IFjp) and the inferior frontal gyrus (FOP3, FOP4). For NON-SYMBOLIC MAGNITUDE, the IPS (IP0, IP1, IPS1, MIP, right IP2 and AIP) was recruited bilaterally. Moreover, we observed bilateral activation in the inferior frontal cortex (left IFjp, right IFSp) and right activation in the supramarginal gyrus (PFt, PFm), the inferior frontal gyrus (FOP4, FOP5) and the DLPFC (p9-46v). The NON-SYMBOLIC MAGNITUDE > SYMBOLIC MAGNITUDE contrast revealed a preferential activation in secondary areas, namely, the right supramarginal gyrus (PFm; Fig. 5C). No significant activation was obtained for the SYMBOLIC MAGNITUDE > NON-SYMBOLIC MAGNITUDE contrast.

### 3.2 TMS

#### 3.2.1 Symbolic and non-symbolic

**Fig. 6:**
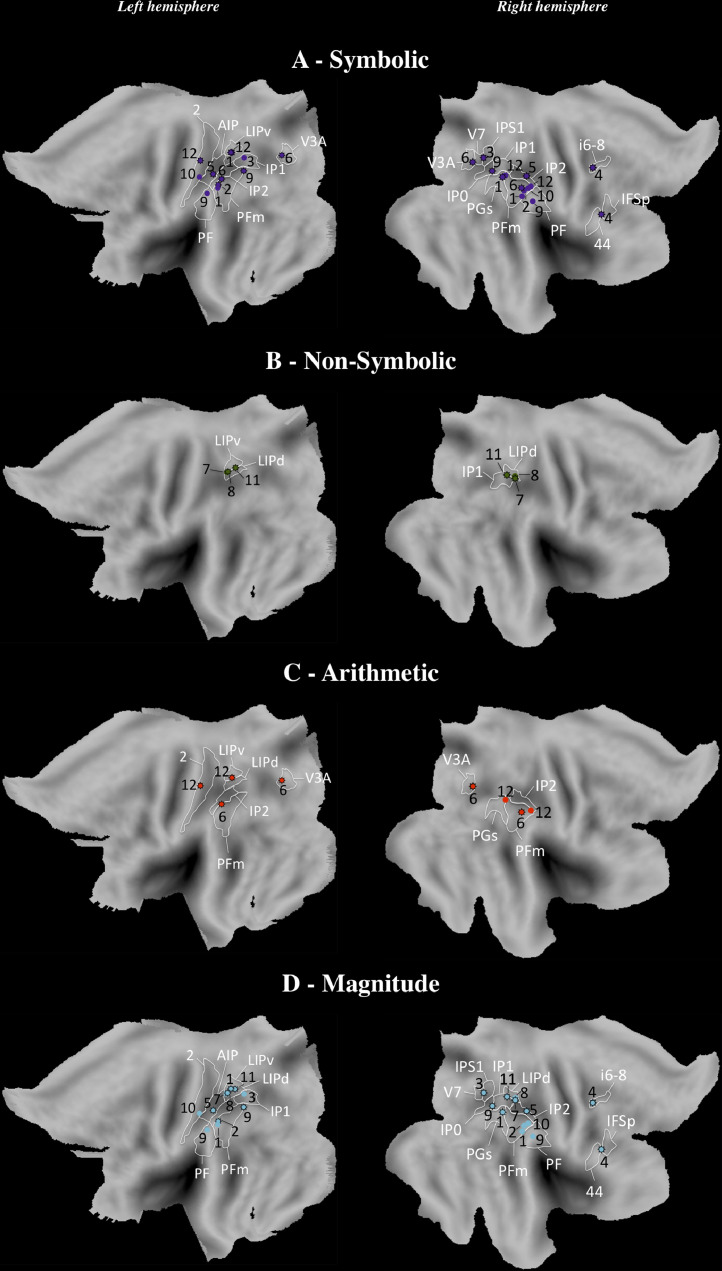
Localizations of stimulation in TMS studies in (A) SYMBOLIC, (B) NON-SYMBOLIC, (C) ARITHMETIC, and (D) MAGNITUDE. Localizations are viewed on PALS-B12 left and right atlas surface configurations (flat maps; Van Essen, 2005). Star: Deficit; Sphere: Normal. The parcellation is based on Glasser et al. (2016). For abbreviations and explanation, see the main text and Appendix.

For SYMBOLIC, the majority of studies revealed that performance was affected by bilateral IPS-TMS (IP1, IP2, left AIP, left area lateral intraparietal ventral [LIPv], right IPS1 and IP0; Fig. 6A). Some studies have shown that a virtual lesion of the inferior parietal lobe (right PGs, left PFm) caused a deficit. However, as can be seen in Fig. 6A, the TMS effect is distributed alongside the IPS (PF-TMS and anterior PFm-TMS did not cause impairment). This is more visible in the right hemisphere. Moreover, we observed a disruption of performance when the TMS was applied over secondary areas such as the right inferior frontal cortex (IFSp, 44) and DLPFC (i6-8), the dorsal stream visual cortex (V3A, right V7) and the left primary somatosensory complex (2). For NON-SYMBOLIC, we found very few studies, but as for SYMBOLIC, bilateral IPS-TMS (area lateral intraparietal dorsal [LIPd], left LIPv, right IP1) disrupted the performance (Fig. 6B).

#### 3.2.2 Arithmetic and magnitude

For ARITHMETIC, in spite of the few studies collected, the performance was more affected by left IPS-TMS (LIPv, LIPd, IP2) than right IPS-TMS. In both hemispheres, we noticed that TMS over the supramarginal gyrus (PFm) and the dorsal stream visual cortex (V3A) caused impairment. Moreover, the disruption of the left primary somatosensory complex (2) affected the performance (Fig. 6C). For MAGNITUDE, we observed a pattern similar to SYMBOLIC, namely, bilateral IPS-TMS (LIPd, left LIPv and AIP, right IP0, IP1, IP2 and IPS1) and TMS alongside the IPS (left PFm, right PGs) caused a deficit. Moreover, in the right hemisphere, the performance was impaired by TMS over the inferior frontal cortex (IFSp, 44) and the DLPFC (i6-8) (Fig. 6D). Because of the very small numbers of studies for SYMBOLIC MAGNITUDE and NON-SYMBOLIC MAGNITUDE, we did not report findings in these two conditions as done above for neuroimaging and below for brain-damaged patients.

### 3.3 Brain-damaged patients

Remember that we could not perform a quantitative meta-analysis. The results presented here are descriptive and, as a result, have to be taken with caution. These results are based on point-scores (difference between the percent score of the patient group and that of the matched control group). The lower the point-score, the more the patient group was impaired compared to the control group.

#### 3.3.1 Symbolic and non-symbolic

**Fig. 7:**
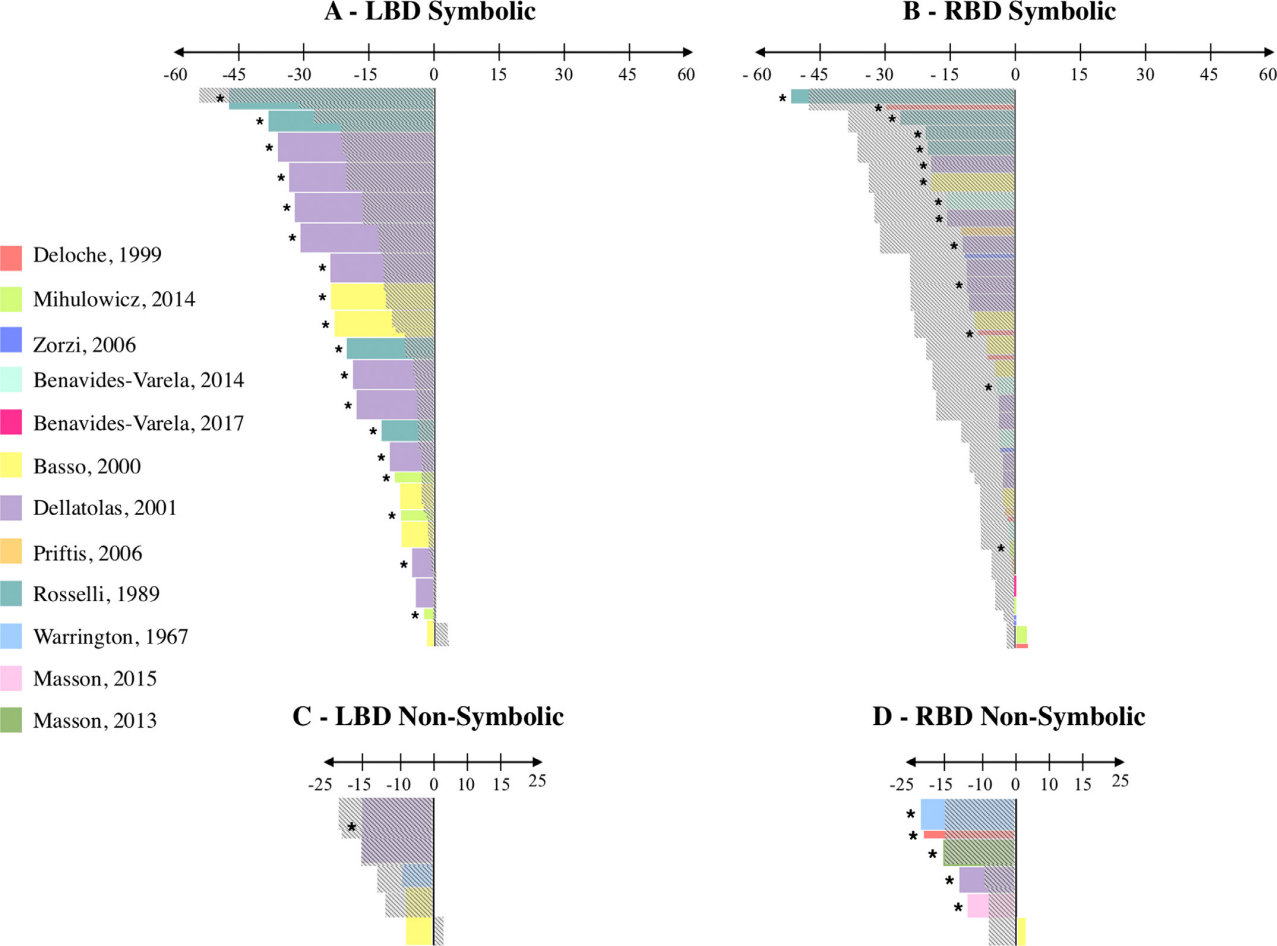
SYMBOLIC and NON-SYMBOLIC (Brain-damaged patients). Each color represents a study. The horizontal axis depicts the value of the point-score; Negative: Patient group 〈 Control group; 0: Patient group = Control group; Positive: Patient group 〉 Control group. The number of patients for each study is represented by the height of the rectangle: The higher the rectangle, the greater the number of patients in the study. Stars indicate significant differences between the patient group and the control group (information based on the original paper). For each condition (SYMBOLIC and NON-SYMBOLIC), we superposed in grey the area covered by the point-scores of LBD patients on the points scores of RBD patients, and vice versa, in order to facilitate the comparison of performance between LBD and RBD patients.

Results for SYMBOLIC are shown in Fig. 7A and Fig. 7B. As can be seen, LBD patients performed significantly worse than their matched controls in 86% of the tasks included (19/22) whereas RBD performed significantly worse than their matched controls in only 35% of the tasks included (14/40). Better performance of RBD patients over LBD patients was confirmed by point-scores, which were generally lower in LBD patients than in RBD patients. A nearly opposite pattern was observed for NON-SYMBOLIC in that LBD performed significantly worse than their matched controls in 33% (1/3; Fig. 7C) and RBD patients in 83% (5/6; Fig. 7D) of the tasks included. Point-scores were also higher in LBD patients than in RBD patients.

#### 3.3.2 Arithmetic and magnitude

**Fig. 8:**
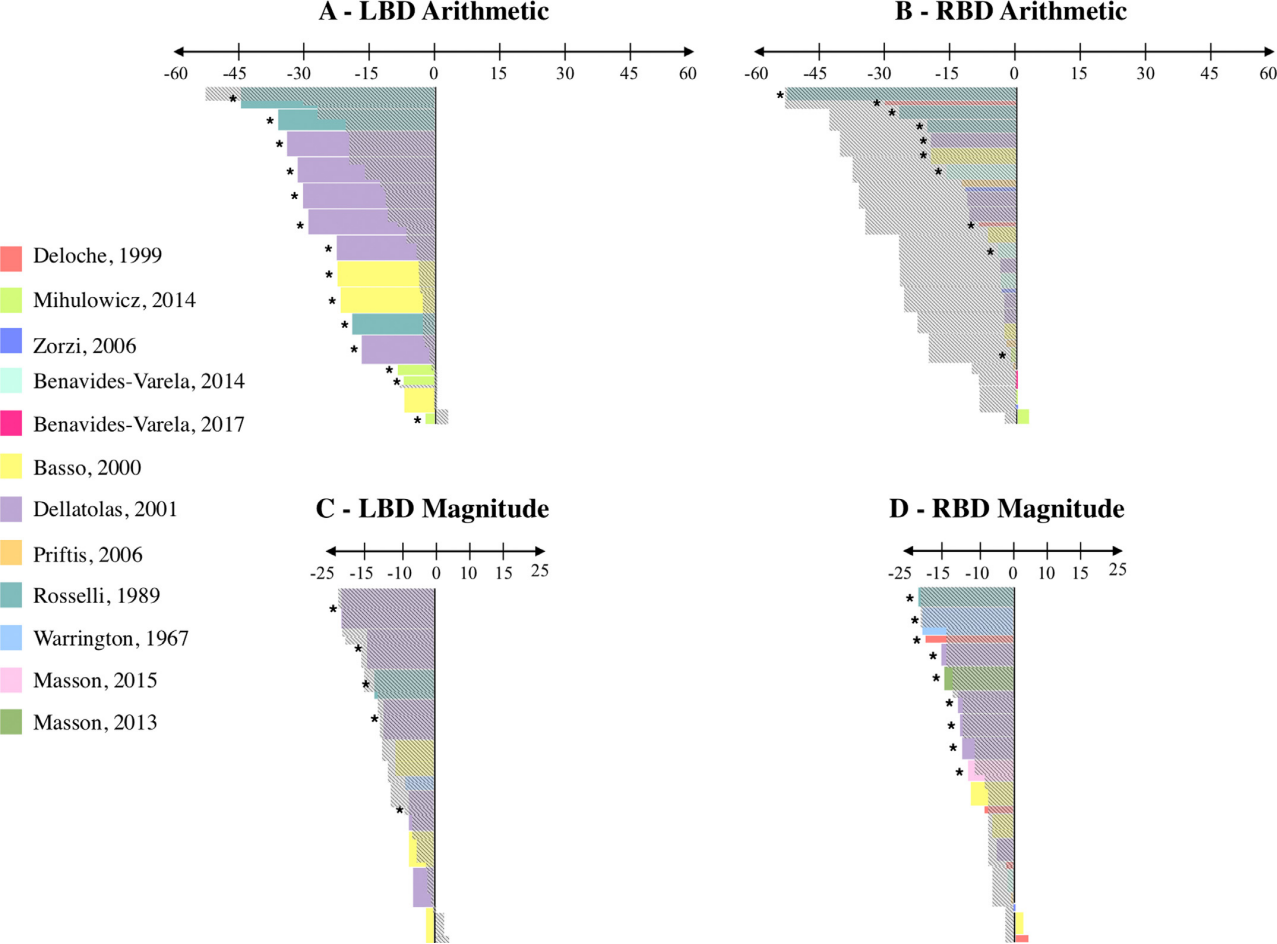
ARITHMETIC and MAGNITUDE (Brain-damaged patients). Each color represents a study. The horizontal axis depicts the value of the point-score; Negative: Patient group 〈 Control group; 0: Patient group = Control group; Positive: Patient group 〉 Control group. The number of patients for each study is represented by the height of the rectangle: The higher the rectangle, the greater the number of patients in the study. Stars indicate significant differences between the patient group and the control group (information based on the original paper). For each condition (ARITHMETIC and MAGNITUDE), we superposed in grey the area covered by the point-scores of LBD patients on the points scores of RBD patients, and vice versa, in order to facilitate the comparison of performance between LBD and RBD patients.

Results for ARITHMETIC are illustrated in Fig. 8A and Fig. 8B. LBD patients performed significantly worse than their matched controls in 93% of the tasks included (14/15), whereas RBD performed significantly worse than their matched controls in only 37% of the tasks included (10/27). Better performance of RBD patients over LBD patients was confirmed by point-scores, which were generally lower in LBD patients than in RBD patients. A different pattern was found for MAGNITUDE in that no clear difference was observed between LBD (50%; 5/10 of the tasks included; Fig. 8C) and RBD patients (47%; 9/19 of the tasks included; Fig. 8D). Point-scores were also relatively similar between LBD and RBD patients.

#### 3.3.3 Symbolic and non-symbolic magnitude

**Fig. 9:**
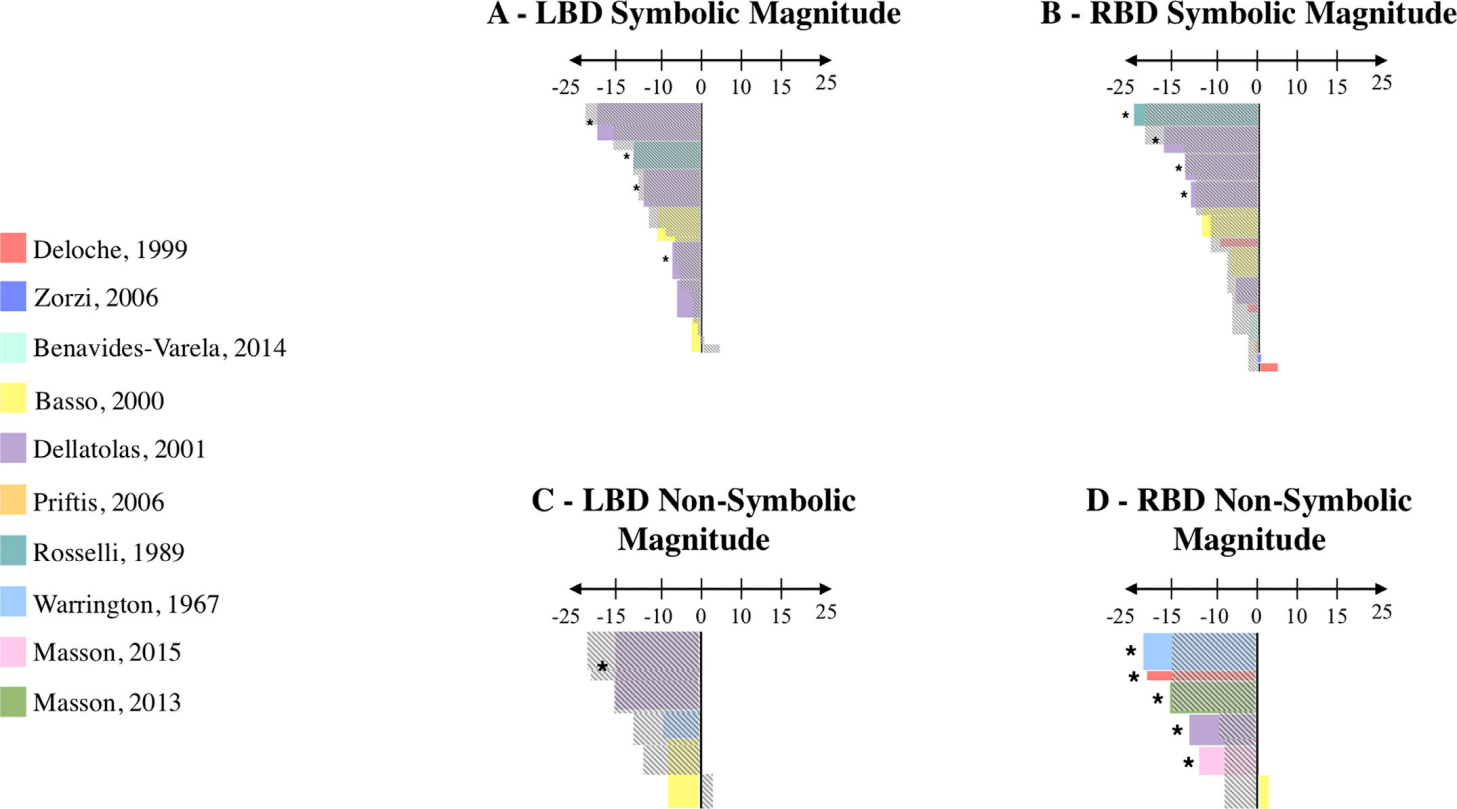
SYMBOLIC MAGNITUDE and NON-SYMBOLIC MAGNITUDE (Brain-damaged patients). Each color represents a study. The horizontal axis depicts the value of the point-score; Negative: Patient group 〈 Control group; 0: Patient group = Control group; Positive: Patient group 〉 Control group. The number of patients for each study is represented by the height of the rectangle: The higher the rectangle, the greater the number of patients in the study. Stars indicate significant differences between the patient group and the control group (information based on the original paper). For each condition (SYMBOLIC MAGNITUDE and NON-SYMBOLIC MAGNITUDE), we superposed in grey the area covered by the point-scores of LBD patients on the points scores of RBD patients, and vice versa, in order to facilitate the comparison of performance between LBD and RBD patients.

Results for SYMBOLIC MAGNITUDE are shown in Fig. 9A and Fig. 9B. LBD patients performed significantly worse than their matched controls in 57% of the tasks included (4/7), whereas RBD performed significantly worse than their matched controls in only 31% of the tasks included (4/13). However, better performance of RBD patients over LBD patients was not confirmed by point-scores, which were relatively similar between LBD and RBD patients. For NON-SYMBOLIC MAGNITUDE, LBD patients performed significantly worse than their matched controls in 33% of the tasks included (1/3; Fig. 9C), whereas RBD performed significantly worse than their matched controls in 83% of the tasks included (5/6; Fig. 9D). The greater difficulties for RBD patients in NON-SYMBOLIC MAGNITUDE were supported by point-scores.

## 4 Discussion

Our goal was to test the main predictions derived from the TCM, leading us to focus on the hypothesis that the representation of numerical quantities rests on a single format-independent representation (i.e., the analogical code) involving both IPS. We were also interested in the link between arithmetic facts and left AG (i.e., the auditory-verbal code). We will begin by discussing these two aspects in light of the neuroimaging and TMS findings. As mentioned, these findings seem to generally confirm the predictions derived from the TCM, even if some refinement is needed. Then, we will address the issue of inter-hemispheric compensation based on data from brain-damaged patients in order to explain the diverging results between neuroimaging and brain-damaged patients studies.

### 4.1 Main predictions of the TCM: neuroimaging and TMS

#### 4.1.1 The analogical code (IPS)

A strong prediction from the TCM is that both IPS possess an analogical and format-independent representation of numerical quantities (i.e., the analogical code). This format-independent hypothesis of IPS has been challenged based on evidence indicating different cerebral correlates for symbolic versus non-symbolic formats (e.g., Ansari, 2007; Cantlon et al., 2009; Piazza et al., 2007; Sasanguie et al., 2017; Venkatraman et al., 2005). This has led some authors to formulate alternative hypotheses, such as the format-dependent processing hypothesis or the multiple representations hypothesis, according to which each (or at least one of) IPS might possess format-dependent representations (Cohen Kadosh and Walsh, 2009; Cohen Kadosh et al., 2007, 2011; see also Holloway et al., 2010; Sokolowski et al., 2017). The main finding from neuroimaging studies reported here is a bilateral activation of IPS in all conditions, whatever the format (SYMBOLIC or NON-SYMBOLIC) or the task (ARITHMETIC or MAGNITUDE). TMS studies corroborated this pattern, indicating that the majority of IPS stimulations disrupted the performance in SYMBOLIC and NON-SYMBOLIC. Taken together, these findings go against any format-dependent hypothesis of IPS and validate the format-independent hypothesis of IPS derived from the TCM. The IPS being a large region, an outstanding issue is to identify which sub-areas of the IPS are specifically involved in the analogical code (e.g., neuroimaging studies indicated an activation of the right AIP in SYMBOLIC but not in NON-SYMBOLIC). Note also that our results diverge from those reported in a recent meta-analysis of neuroimaging studies by Sokolowski et al. (2017). Indeed, our SYMBOLIC > NON-SYMBOLIC and NON-SYMBOLIC > SYMBOLIC contrasts did not reveal specific activations within the parietal cortex contrary to the results described by these authors. The best explanation for this discrepancy is the difference in terms of threshold employed in each meta-analysis (50 mm^3^ in Sokolowski et al., 2017, namely, a very liberal threshold; 100 mm^3^ in the present meta-analysis, namely, a more conservative threshold) and the inclusion criteria of tasks.^2^

**Fig. 10:**
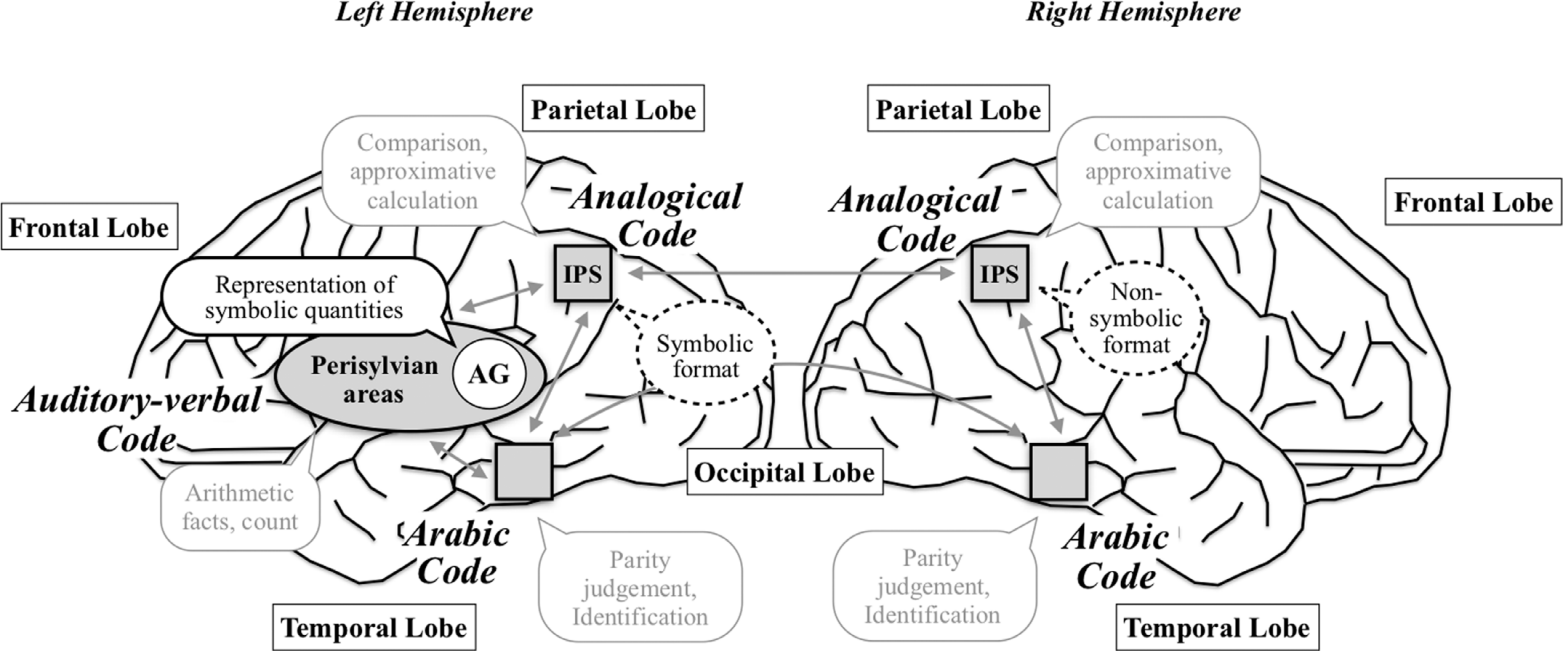
Updated version of the TCM based on our key findings. This figure is based on Dehaene and Cohen (1995). The neuroanatomical implementation of the three numerical codes remains unchanged. Based on neuroimaging studies, we add a new role for the left AG, namely, matching quantities to Arabic digits. Based on brain-damaged patients studies, we also suggest that, even if both IPS possess an analogical, format-independent representation of numerical quantities (i.e., analogical code), the left IPS might show a preference for symbolic formats and the right IPS for non-symbolic formats.

The TCM does not predict an involvement of the left AG in the analogical code even in simple symbolic comparison tasks where Arabic digits are employed. However, the meta-analyses conducted from neuroimaging studies showed a quasi-systematic activation of this brain area in all conditions (except for NON-SYMBOLIC MAGNITUDE). In other words, our findings do not corroborate this prediction, leading us to propose a refinement of the TCM based on a recent hypothesis called the symbol-to-referent mapping hypothesis (Holloway et al., 2010; see also Grabner et al., 2007, 2013; Price and Ansari, 2011; see Fig. 10). This hypothesis posits that the left AG is critical to link visual symbols to their quantitative referents. In other words, the left AG might participate in identifying and making sense of Arabic digits, by matching quantities to Arabic digits.

#### 4.1.2 The auditory-verbal code (Left AG)

The TCM predicts that the left AG and perisylvian areas are involved in the auditory-verbal code, enabling the retrieval of arithmetic facts from long-term memory. Results from neuroimaging studies indicate that the left AG and the left middle temporal gyrus (MTG) were activated in both SYMBOLIC and ARITHMETIC, thereby confirming the aforementioned prediction.^3^ The key issue is to explain the involvement of these regions in the retrieval of arithmetic facts. The link between phonological skills and arithmetic facts has been clearly drawn in the literature (e.g., Koponen et al., 2013; Schleepen et al., 2016; Vukovic and Lesaux, 2013). More specifically, arithmetic facts might be stored in a phonological format because of rote learning during childhood. Thus, in children, poor phonological skills might explain arithmetic fact retrieval difficulties (e.g., De Smedt, 2018). The involvement of phonological skills in the retrieval of arithmetic facts has also been demonstrated in adults. For instance, adults can meet difficulties to retrieve arithmetic facts (e.g., multiplication tasks) in a phonological suppression paradigm (Lee and Kang, 2002). Therefore, the left AG and MTG might play a key role for phonological skills critical to the retrieval of arithmetic facts, as suggested by the TCM (Dehaene, 1992).

#### 4.1.3 Other regions of interest

##### 4.1.3.1 SMG

The role played by each SMG in numerical cognition might differ. More specifically, neuroimaging studies indicate that the left SMG is activated in SYMBOLIC but not NON-SYMBOLIC. Activation is also found in ARITHMETIC and MAGNITUDE, but only for SYMBOLIC MAGNITUDE, and not for NON-SYMBOLIC MAGNITUDE. Results from TMS studies are broadly consistent, showing that stimulation of the left SMG disrupts performance on SYMBOLIC and ARITHMETIC. In broad terms, the left SMG seems to be engaged in numerical tasks involving arithmetic facts and symbolic formats. Fulbright et al. (2003) reported that the left SMG was activated in accord with the distance effect for numbers, suggesting a potential role for this region in the mental line number. Although this interpretation remains possible, it is inconsistent with our results, which stress a clear link between the left SMG and arithmetic facts. Therefore, the outstanding issue is to specify the cognitive processes underlying by the left SMG in the context of numerical cognition. Concerning the right SMG, neuroimaging results are less clear-cut, highlighting an activation of this brain area in all conditions except SYMBOLIC MAGNITUDE. Again, TMS results reported a somewhat similar pattern. Göbel et al. (2001b) proposed that the right SMG might support small number representations. This proposal is however at odds with our findings given that we observed an association between the right SMG and NON-SYMBOLIC MAGNITUDE, implying the processing of large quantities as well as approximation. So, as for the left SMG, the role played by the right SMG in numerical cognition is an open question.

##### 4.1.3.2 IFC

We observed a bilateral activation of the IFC in almost all conditions (neuroimaging studies). In TMS studies, only one study stimulated the right IFC, reporting a disrupted performance during a symbolic magnitude task (4: Rusconi et al., 2009). Based on these findings, the issue is whether IFC is directly involved in numerical skills or not (e.g., the left IFC because of its link with language, see Glasser et al., 2016; the right IFC because of its link with inhibition, executive control and working memory, see Aron et al., 2014; Ischebeck et al., 2009; Song and Jiang, 2006).

##### 4.1.3.3 IFG

Neuroimaging studies revealed a quasi-systematic activation of the left IFG in all conditions (except for NON-SYMBOLIC MAGNITUDE). This suggests that this brain area might participate in the retrieval of arithmetic facts. However, its role might differ from that of the left AG or MTG, by supporting quantity-based computations and not automatic retrieval of arithmetic facts. Consistent with this, it has been shown that the left IFG was preferentially activated when participants had to solve untrained rather than trained problems (e.g., Bloechle et al., 2016; Venkatraman et al., 2006) and less and less recruited in the retrieval of arithmetic facts over the development (Qin et al., 2015; Prado et al., 2014). Concerning the right IFG, neuroimaging studies revealed an activation of this brain area in NON-SYMBOLIC and MAGNITUDE as well as a preferential activation for the NON-SYMBOLIC > SYMBOLIC contrast. One possible interpretation is that the right IFG is not a key region for numerical cognition, but rather a “non-specific” region supporting inhibitory control as proposal by Aron et al. (2014). In this perspective, inhibitory control might be more pronounced in non-symbolic tasks in which a certain form of incongruency occurs between the area occupied by the dots and the number of dots to be estimated. Thus, the right IFG might be activated in order to inhibit the salient feature (i.e., the area occupied by the dots) in favor of the relevant feature for the task (i.e., the number of dots; see Gilmore et al., 2013).

##### 4.1.3.4 Motor and premotor areas

Neuroimaging studies also indicated bilateral activation of motor and premotor areas in all conditions. Given the importance of these brain areas for hand and finger movements, this bilateral activation could notably reflect finger-counting strategies developed during childhood (Andres et al., 2008; Dormal et al., 2012a; Pesenti et al., 2000).

### 4.2 Brain-damaged patients

#### 4.2.1 No inter-hemispheric compensation

Our meta-analysis stressed that LBD patients scored lower than RBD patients in SYMBOLIC and ARITHMETIC. In agreement with the TCM, a significant proportion of LBD patients could have lesions of AG and perisylvian areas, causing arithmetic fact retrieval difficulties. In addition, we found that LBD and RBD patients did not clearly differ in SYMBOLIC MAGNITUDE, suggesting the presence of inter-hemispheric compensation as predicted again by the TCM. All our results were nevertheless not fully consistent with the predictions derived from the TCM. Specifically, we observed that RBD patients met more difficulties than LBD patients in NON-SYMBOLIC MAGNITUDE. A potential interpretation is that, before the acquisition of symbolic representations, non-symbolic magnitude is supported by the right IPS (Ansari, 2016; see also Hyde et al., 2010; Izard et al., 2008). Then, when the expertise for symbolic numbers increases, symbolic magnitude shifts to the left IPS. In other words, the processing of non-symbolic magnitude might preferentially recruit the right IPS, explaining why RBD patients cannot compensate by using the left hemisphere. This format-dependent interpretation is at odds with predictions from the original version of the TCM (Fig. 10).

#### 4.2.2 Opposite results between neuroimaging/TMS and brain-damaged patients

One of the more intriguing findings from the meta-analyses conducted here is discrepant results between obtained from neuroimaging/TMS (i.e., bilateral activation of IPS) and brain-damaged patients (i.e., right hemispheric lateralization for non-symbolic formats). The discrepancy is difficult to interpret. A potential interpretation is based on the age difference between healthy participants in neuroimaging and TMS studies (about 26 years old) and brain-damaged patients (about 54 years old). In line with this, Huang et al. (2012) showed an age-related distribution of parietal activation for a symbolic comparison task. The young adults activated the right parietal cortex, whereas elderly adults engaged both the left and the right parietal cortex. Unfortunately, these findings cannot explain the discrepancy reported here because we observed the opposite pattern: Bilateral involvement of IPS in neuroimaging and TMS studies (young participants) and a performance depending on the lesion side in brain-damaged patients (older participants). Another interpretation can be offered based on the neuroimaging results, which highlight that numerical cognition is supported by a bilateral fronto-parietal network (Fig. 2). It has been shown that normal aging is accompanied with more important modifications in the prefrontal lobes than in other brain regions (e.g., West, 1996). So, if the processing of magnitude requires not only the IPS but also a wider network including the prefrontal lobe (e.g., Dehaene et al., 2003; Dehaene and Cohen, 1995, 1997), then any modification in the prefrontal lobes can generate more difficulties to solve numerical tasks. In this way, LBD and RBD patients could have shown specific difficulties depending on the format because of age-related modifications and/or neurological damage in the prefrontal lobes.

## 5 Conclusion

The TCM is the most comprehensive framework of numerical skills. On the whole, our findings corroborate the main predictions of the TCM. Neuroimaging and TMS studies demonstrate a bilateral involvement of the IPS whatever the task and the format, confirming the format-independent hypothesis of IPS suggested by the TCM. We also found that the left AG plays a key role in arithmetic facts. Nevertheless, our findings also stress that some refinements of the TCM are needed to account for all the data reported here. First, the left AG seems to be involved also in magnitude tasks, suggesting that this brain region might participate in identifying and making sense of Arabic digits, by matching quantities to Arabic digits (Holloway et al., 2010; Price and Ansari, 2011). Results from brain-damaged patients also stress that the right hemisphere might be specifically engaged in numerical magnitude. This does not fully challenge the TCM, but suggests specific non-symbolic magnitude representations in the right IPS and general symbolic magnitude representations in the left IPS. Moreover, two lateralized fronto-parietal circuits seem to be associated with specific numerical skills, a right one for non-symbolic magnitude and a left one for symbolic magnitude. Further studies are needed to explore the association fiber pathways between these frontal structures and IPS. It would be also relevant to assess more specifically numerical magnitude disorders in RBD patients, in order to better understand their origins, namely, visual or reflecting a real analogical code deficit. Future studies are also required to determine the role of less prominent structures, such as SMG or IFG. Finally, it appears important to elucidate the discrepancy reported here between neuroimaging/TMS studies and brain-damaged patients studies. The iterative method proposed by Price and Friston (2002) might be useful in this respect, allowing the combination of the lesion-deficit and neuroimaging approaches.

## Figures and Tables

**Table:** 

44: Inferior Frontal Gyrus
46: Dorsolateral Prefrontal cortex
a9-46v: Dorsolateral Prefrontal cortex
AG: Angular Gyrus
AIP: Anterior Intraparietal area
DLPFC: Dorsolateral Prefrontal Cortex
FOP2; FOP3; FOP4; FOP5: Frontal Opercular cortex
i6-8: Dorsolateral Prefrontal cortex
IFG: Inferior Frontal Gyrus
IFja; IFjp; IFsa; IFSp: Inferior Frontal Gyrus
IP0; IP1; IP2: Intraparietal Sulcus
IPS: : Intraparietal Sulcus
IPS1: Intraparietal Sulcus area 1
LIPd: area Lateral Intraparietal dorsal
LIPv: area Lateral Intraparietal ventral
MIP: Medial Intraparietal area
OP2-3: Posterior Opercular cortex
p9-46v: Dorsolateral Prefrontal cortex
PF; PFm; PFt: Supramarginal Gyrus
PGs: Angular Gyrus
pMTG: Middle Temporal Gyrus
rIFG: right Inferior Frontal Gyrus
SFL: Superior Frontal Language area
SMG: Supramarginal Gyrus
TE1p: Middle Temporal Gyrus
V3A; V3B; V6A; V7: Dorsal Stream Visual Cortex

## Appendix B Supplementary materials

Image, application 1

## Footnotes

1 Dehaene et al. (1993) observed that participants, who had to judge the parity of a number (from 0 to 9), responded faster with the left hand than with the right hand when the number was small, and faster with the right hand than with the left hand when the number was large. The numerical magnitude was automatically processed even if it was irrelevant to solving the task. The SNARC effect reported in this study provided evidence that numerical quantity might be represented on a mental number line.

2 We conducted an additional analysis using the same threshold as the one employed by Sokolowski et al. (2017). Results are presented in **Supplementary Material.** This analysis did not modify our results, suggesting that the discrepancy should rather come from the criteria used to include the studies. We tried as much as possible to include “pure” magnitude tasks (i.e., comparison of Arabic digits or non-symbolic stimuli) and arithmetic tasks (i.e., single-digit operations). We excluded all tasks requiring counting or multi-digit mental calculation. So, our inclusion criteria could have been stricter than those used by Sokolowski et al. (2017).

3 As mentioned in the previous section, neuroimaging studies also revealed an activation of the left AG in NON-SYMBOLIC and MAGNITUDE, which is not predicted by the TCM. We will not discuss further this aspect here (see discussion just above).
