# Supplementary material for: Numerical cognition: A meta-analysis of neuroimaging, transcranial magnetic stimulation and brain-damaged patients studies
Source: Neuroimage Clin. 2019 Oct 22;24:102053. doi: 10.1016/j.nicl.2019.102053 (PMC6978218; doi:10.1016/j.nicl.2019.102053)
Supplement: Supplementary file 1 [file mmc1.docx]

### **Supplementary Material**

### Numerical Cognition: A Meta-Analysis of NeuroImaging, Transcranial Magnetic Stimulation and Brain-Damaged Patients studies

Alexandrine Faye^1*^, Sophie Jacquin-Courtois^2,3^, Emanuelle Reynaud^1^, Mathieu Lesourd^4,5^, Jérémy Besnard^6^, and François Osiurak^1,7^

^1^Laboratoire d’Etude des Mécanismes Cognitifs (EA 3082), Université de Lyon, France

^2^Integrative, Multisensory, Perception, Action, & Cognition Team (INSERM-CNRS-UMR 5292), Université de Lyon, France

^3^Mouvement et Handicap, Hospices Civils de Lyon et Centre de Recherche en Neurosciences de Lyon, Hôpital Henry Gabrielle, St Genis Laval, France

^4^Aix Marseille Université, CNRS, LNC, Laboratoire de Neurosciences Cognitives, Marseille, France

^5^Aix Marseille Université, CNRS, Fédération 3C, Marseille, France

^6^Laboratoire de Psychologie des Pays de la Loire (EA 4638), Université de Nantes et d’Angers

^7^Institut Universitaire de France, Paris, France

# Method S1. Selection of studies

### Method S1.1. Neuroimaging studies

We  limited our search to studies published between January 1995  and December 2016. To refine our search for neuroimaging studies, we used the conjunction of the keywords: “imaging”, “functional magnetic  resonance imaging”, “fMRI”, “positron emission tomography”, “PET”, “neuroimaging”, “magnitude”, “analog”, “calculation”, “arithmetic”, “number processing”, “symbolic”, “non-symbolic”, “numerical cognition”, “adults”. A study was included if it comprised a magnitude (e.g., a comparison task) or arithmetic tasks (e.g., an addition task), with symbolic or non-symbolic numbers. From this search, 96 studies returned at the date of 08/12/2016.

We  evaluated relevant studies for inclusion, in accordance with a several  selection criteria:

(1) Reviews and Theoretical papers were ruled out;

(2) Studies had to use functional magnetic resonance imaging or Positron Emission Tomography;

(3) Studies had to include only neurologically healthy and adult participants;

(4) Suitability of the tasks used in the context of our goal. As explained above, we considered both symbolic and non-symbolic number tasks. Moreover, only studies using visual stimuli were included except for one study using auditory stimuli;

(5) Scanning of neuroimaging results had to depend on whole-brain analysis. Therefore, regions of interest analyses were ruled out from our selection;

(6) The final list of activation peaks (i.e., foci) with their coordinates must be related in a stereotactic space (i.e., MNI or Talairach);

(7) We picked only corrected results for multiple comparisons with a statistical significance threshold of *p* < 0.05 or, for a small part of the selected results uncorrected data thresholded at *p* < 0.005. The same threshold must be used uniformly throughout the whole brain. Results developed from ROI (Region of Interest) or SVC (Small Volume Correction) analyses were ruled out even though spatial coordinates were supplied. It was important to avoid experimenter-induced bias in the locations at which effects could be singled out, because our meta-analytic statistical tests supposed that foci were spatially randomly distributed throughout the whole brain under the H0 assumption.

This evaluation resulted in 28 studies  and 45 experiments meeting our criteria, involving a total of 394 participants (all right-handed) and 479 peaks of activation (the participants who carried out more than one experiment were only  counted once). Table S1 (Below) described the detail of the included studies.

### Method S1.2. TMS studies

We  restricted our search to studies published between October 2001  and March 2017. For identifying relevant studies, we used the conjunction of the keywords: “transcranial magnetic stimulation”, “TMS”, “rTMS”, “magnitude”, “analog”, “calculation”, “arithmetic”, “number processing”, “symbolic”, “non-symbolic”, “numerical cognition”, “adults”. In the same way as for the neuroimaging studies, a study was included if it comprised a magnitude (e.g., a comparison task) or arithmetic tasks (e.g., an addition task), with symbolic or non-symbolic numbers. This search identified 20 studies at the date of 31/03/2017. Then, we restricted the studies to include according to several selection criteria:

(1) Studies had to use TMS stimulations (i.e., single-pulse, double-pulse or repetitive). Studies with continuous Theta-Burst Stimulations (cTBS) were also included;

(2) They comprised neurologically healthy and adults participants;

(3) Stimulations produced on left and right hemispheres were reported;

(4) Stimulation coordinates must be related in a stereotactic plane (i.e., MNI or Talairach).

This evaluation led to the selection of 12 studies including 150 healthy controls. We reported 32 brain stimulations (15 left and 17 right). The included studies are described in more detail in Table S2 (Below).

### Method S1.3. Patient studies

We  limited our search to studies published between October 1967  and January 2017. For identifying pertinent studies, the following keywords were used: “magnitude”, “analog”, “calculation”, “arithmetic”, “number processing”, “symbolic”, “non-symbolic”, “numerical cognition”, “parietal lobe”, “IPS”, “intraparietal sulcus”, “cerebral lesions”, “patients”, “brain damaged”. In the same way as for the neuroimaging and TMS studies, a study was included if it comprised a magnitude (e.g.*,* a comparison task) or arithmetic tasks (e.g., an addition task), with symbolic or non-symbolic numbers. This search returned 117 studies at the date of 30/01/2017. We selected brain-damaged patients’ studies depending on several  selection criteria:

(1) Only studies presenting patients with a unilateral lesion due to stroke or tumor (LBD or RBD) were included;

(2) Single case studies were not considered;

(3) Patients must be compared to a control group of healthy subjects;

(4) Studies had to provide the raw score and the maximal score of the patients and control groups.

This final assessment resulted in 12 studies including 188 LBD patients and 259 RBD patients. We reported 25 scores for LBD patients and 46 scores for RBD patients. The detail of these studies is described in Table S3 (Below).

Table S1. Summary of neuroimaging studies

| Author (year) | Subjects’ age (mean (sd) or range) | N | Method | Tasks | | Control condition |
| --- | --- | --- | --- | --- | --- | --- |
|  |  |  |  | ARITHMETIC | MAGNITUDE |  |
| Attout et al. (2014) | 20,9; 18-28 | 26 | fMRI |  | Numerical order judgment (S) | Luminance judgment |
| Cappelletti et al. (2009b) | 54,6; 20-62 | 22 | fMRI |  | Conceptual number tasks (S)  Quantity number tasks (S and NS) | Fixation |
| Chassy and Grodd (2012) | 28,62 (5,75) | 16 | fMRI |  | Dot comparison (NS)  Positive Arabic digit comparison (S)  Negative Arabic digit comparison (S) | Baseline activity was subtracted from task conditions |
| Chochon et al. (1999) | 20-30 | 8 | fMRI | Multiplication (S)  Subtraction (S) | Arabic digit comparison (S) | Letter naming |
| Delazer et al. (2003) | 30.5 (4.8) | 13 | fMRI | Simple multiplication (S)  Compound multiplication (S) |  | Number matching |
| Dormal et al. (2010) | 21 (2,3) | 15 | fMRI |  | Categorization of linear arrays of dot presented simultaneously (NS) Categorization of sequences of dot (NS) Conjunction of the both tasks (NS) | Color detection |
| Dormal et al. (2012a) | 21 (2,3) | 15 | fMRI |  | Dot categorization (NS) | Color detection |
| Dormal et al. (2009) | 21 (2,3) | 14 | fMRI |  | Dot comparison (NS) | Color detection |
| Fias et al. (2003) | 23.1; 21-29 | 18 | fMRI |  | Arabic digit comparison (S) | Dimming task |
| Gruber et al. (2001) | 25.8 (2,9) | 6 | fMRI | Simple calculation (multiplication and division) (S)  Compound calculation (multiplication and division) (S) |  | Number substitution  Letter substitution  Letter pseudo-multiplication  Letter pseudo-division |
| Hayashi et al. (2013) | 19-30 | 26 | fMRI |  | Dot comparison (NS) | To press a button with finger |
| Knops and Willmes (2014) | 24,9; 20-31 | 17 | fMRI | Addition (S)  Subtraction (S) |  | Low-level baseline condition for 20,000 ms (German word for break (“Pause”) |
| Leroux et al. (2009) | 23,2 (1,9) | 9 | fMRI |  | Numerosity comparison (NS) | Color detection |
| Menon et al. (2000) |  | 16 | fMRI | Mental calculation (6 seconds design, 3-operands) (S)  Mental calculation (6 seconds design, 2-operands) (S)  Mental calculation (3 seconds design, 3-operands) (S)  Mental calculation (6 seconds design, 2-operands) (S) |  | Detection of “zero” |
| Pesenti et al. (2000) | 21-29 | 8 | PET | Addition (S) | Arabic digit comparison (S) | Orientation judgment on (non) numerical stimuli |
| Piazza et al. (2004) | 23 | 12 | fMRI |  | Dot fixation (Habituation design) (NS) | Dot fixation (Deviant design) |
| Piazza et al. (2006) | 23-31 | 10 | fMRI |  | Visual and auditory comparison (NS) | Matching task |
| Pinel et al. (1999) | 25.7 | 11 | fMRI |  | Arabic digit and number-word comparison (S) | Letters fixation |
| Pinel et al. (2004) | 23,7 | 15 | fMRI |  | Arabic digit comparison (S) | Number size comparison |
| Rickard et al. (2000) | 24 (20-34) | 8 | fMRI | Multiplication verification (S) |  | Detection of “1” |
| Stanescu-Cosson et al. (2000) | 22-26 | 7 | fMRI | Addition (S) |  | Letter-matching |
| Skagerlung et al.  (2016) | 24,33 (2,41) | 24 | fMRI |  | Dot comparison (NS) | Color detection |
| Venkatraman et al. (2005) | 20-25 | 10 | fMRI | Exact addition (S)  Approximate addition (S)  Exact addition (NS)  Approximate addition (NS) |  | Number matching |
| Vogel et al. (2013) | 18-33 | 14 | fMRI |  | Number positioning on a scale (S) | To move the trackball to a location on the line indicated by an arrow, and to click on it |
| Wei et al. (2014) | 20 (20,6); 18,8-22,5 |  | fMRI |  | Semantic distance judgment with (S: Arabic digit and number-word and NS: dot) | Fixation |
| Zago et al. (2001) | 21 (1) | 6 | PET | Multiplication (S) |  | Reading Arabic digits aloud |
| Zago et al. (2008) | 20-27 | 14 | fMRI | Addition (S) |  | Maintenance of number |
| Abbreviations: sd, standard deviation; N, number of subjects; fMRI, functional Magnetic Resonance Imaging; PET, Positron Emission Tomography; S, Symbolic; N, Non-Symbolic. | | | | | | |

Table S2. Summary of TMS studies

| Number of study: Author (year) | Subjects’ age  (sd or range) | N | Method | Tasks | | TMS Effect | Talairach coordinates  of the stimulation sites | |
| --- | --- | --- | --- | --- | --- | --- | --- | --- |
|  |  |  |  | ARITHMETIC | MAGNITUDE |  | L | R |
| 5: Cappelletti et al. (2009a) | 22.2 (22-23) | 6 | rTMS |  | Arabic digit comparison (S) | The performance was affected by the IPS stimulation but left IPS-TMS inducing a larger impairment than right IPS-TMS | IPS: -42; -38; 40 | IPS: 38; -41; 38 |
| 3: Cohen-Kadosh et al. (2007a) | 28.6 (4.5) | 5 | rTMS |  | Arabic digit comparison (S) | The performance was impaired during disruption of right IPS | IPS: -25; -59; 44 | IPS: 22; -68; 39 |
| 8: Dormal et al. (2008) | 22 (2.7) | 15 | rTMS |  | Dot comparison (NS) | Significant impairment after the left IPS stimulation | IPS: -39; -49; 50 | IPS: 36; -51; 50 |
| 7: Dormal et al. (2012b) | 24 (5) | 10 | rTMS |  | Dot categorization (NS) | Significant impairment when the right IPS was stimulated | IPS: -37; -48; 50 | IPS: 36; -50; 48 |
| 12: Göbel et al. (2006) | 24 (19-40) | 14 | rTMS | Mental addition (S) |  | The performance was affected when rTMS applied over the left IPL | aIPL: -45; -40; 58  pIPL: -40; -61; 54 | aIPL: 45; -40; 58  pIPL: 40; -61; 54 |
| 1: Göbel et al. (2001a) | 25.7 (20-38) | 9 | rTMS |  | Numerical order judgment (S) | rTMS over the AG disrupted the performance | AG: -42; -58; 52 | AG: 42; -58; 52 |
|  | 22.7 (19-31) | 6 | rTMS |  |  | No effect when the SMG was disrupted | SMG: -52; -46; 44 | SMG: 52; -46; 44 |
| 11: Lecce et al. (2015) | 26.7 (19-40) | 14 | cTBS |  | Dot comparison (NS) | The performance was impacted during the IPS-TBS | IPS: -36; -60; 52 | IPS: 28; -56; 52 |
| 6: Salillas et al. (2012) | 23 (21-26) | 12 | Single-pulse TMS | Mental calculation (S) |  | rTMS over the IPS affected the performance | hIPS: -43; -48; 47  vIPS: -24; -76; 30 | hIPS: 43; -48; 47  vIPS: 24; -76; 30 |
| 2: Sandrini et al. (2004) | 28.4 (25-32) | 9 | rTMS |  | Arabic digit comparison (S) | Significant impairment during rTMS over left IPL | IPL: -48; -47; 52 | IPL: 48; -47; 52 |
| 10: Sasanguie et al. (2013) | 22 (4.2) | 17 | rTMS |  | Numerical order judgment (arabic digit and number-word) (S) | No effect when the IPS was disrupted | IPS: -44; -29; 45 | IPS: 48; -39; 45 |
| 4: Rusconi et al. (2009) | 30.1 | 10 | rTMS |  | Numerical order judgment (S) | The SNARC effect was eliminated when rTMS applied over the right IFG and FEF |  | FEF: 31; -1; 47  IFG: 48; 22; 23 |
| 9: Rusconi et al. (2007) | 28.5 | 16 | rTMS |  | Parity judgment (S) | The SNARC effect was eliminated by rTMS over the pPPL | aPPL: -60; -32; 44  pPPL: -36; -64; 36 | aPPL: 60; -32; 44  pPPL: 36; -64; 36 |
| Abbreviations: sd, standard deviation; N, number of subject; S, Symbolic; N, Non-Symbolic; L, Left; R, Right; rTMS, repetitive Transcranial Magnetic Stimulation; IPS, Intraparietal Sulcus; aIPL/pIPL, anterior/posterior Inferior Parietal Lobe; AG, Angular Gyrus; SMG, Supramarginal Gyrus; hIPS/vIPS, horizontal/ventral Intraparietal Sulcus; SNARC, Spatial Numerical Association of Response Code; FEF, Frontal Eye Fields; IFG, Inferior Frontal Gyrus; aPPL/pPPL, anterior/posterior Posterior Parietal Lo  be. | | | | | | | | |

Table S3. Summary of brain-damaged patients studies

| Author (year) | Patients’ age  (sd; range) | N | Site of lesion | Etiology | Tasks | |
| --- | --- | --- | --- | --- | --- | --- |
|  |  |  |  |  | ARITHMETIC | MAGNITUDE |
| Basso et al. (2000) | 58.9 (12.9; 26-75)  62.3 (12; 28-75) | 50  26 | L  R | Stroke | Mental calculation (S)  Written calculation (S)  Estimation of the result of an operation (S) | Perceptive estimation of quantity (NS)  Arabic digit comparison (S)  Number positioning on a scale (S) |
| Benavides-Varela et al. (2014) | 64 (10.6; 42-75) | 24 | R | Stroke | Oral addition (S)  Oral subtraction (S)  Oral multiplication (S) | Arabic digit comparison (S) |
| Benavides-Varela et al. (2017) | 60 (13.3; 29-85) | 30 | R | Stroke | Mental calculation (S) |  |
| Dellatolas et al. (2001) | 50.6 (13.4; 22-75)  51.6 (10.6; 29-69) | 56  24 | L  R | Stroke | Mental calculation (spoken numbers)  Mental calculation (Arabic numbers)  Written addition (S)  Written subtraction (S)  Written multiplication (S)  Estimation of the result of an operation (S) | Perceptive estimation of quantity (NS)  Written number positioning on a scale (S)  Oral number positioning on a scale (S)  Arabic digit comparison (Arabic digit code)  Arabic digit comparison (written verbal code) |
| Deloche et al. (1999) | 24.9 (7.5; 18-39) | 8 | R | Stroke | Mental calculation (S)  Written calculation (S) | Perceptive estimation of quantity (NS)  Number positioning on a scale (S)  Oral Arabic digit comparison (S)  Written Arabic digit comparison (S) |
| Masson et al. (2013) | 59 (11; 35-80) | 25 | R | Stroke; HT |  | Dot comparison (NS) |
| Masson et al. (2015) | 59.3 (11; 40-80) | 22 | R | Stroke; Tumor |  | Dot comparison (NS) |
| Mihulowicz et al. (2014) | 61.6 (16.1)  61 (14) | 21  24 | L  R | Stroke | Oral addition (S)  Oral subtraction (S)  Oral multiplication (S) |  |
|  |  |  |  |  |  |  |
| Priftis et al. (2006) | 65.9 (4.2; 60-72) | 12 | R | Stroke | Oral addition (S)  Oral subtraction (S)  Oral multiplication (S) | Arabic digit comparison (S) |
| Rosselli and Ardila (1989) | 41.43 (13.9; 16-65)  41.43 (13.9; 16-65) | 41  21 | L  R | HT; Tumor | Mental calculation (S)  Written calculation (S)  Complex written operation (S) | Arabic digit comparison (S) |
| Zorzi et al. (2006) | 62 (10.1; 39-70) | 8 | R | Stroke | Oral addition (S)  Oral subtraction (S)  Oral multiplication (S) | Arabic digit comparison (S) |
| Warrington and James (1967) | 41 (15.8; 17-73)  46.7 (15.5; 17-69) | 20  29 | L  R | Stroke; Tumor; Abscess |  | Perceptive estimation of quantity (NS) |
| Abbreviations: sd, standard deviation; N, number of patients; L, Left; R, Right, S, Symbolic; N, Non-Symbolic; HT, Head Trauma. | | | | | | |

*
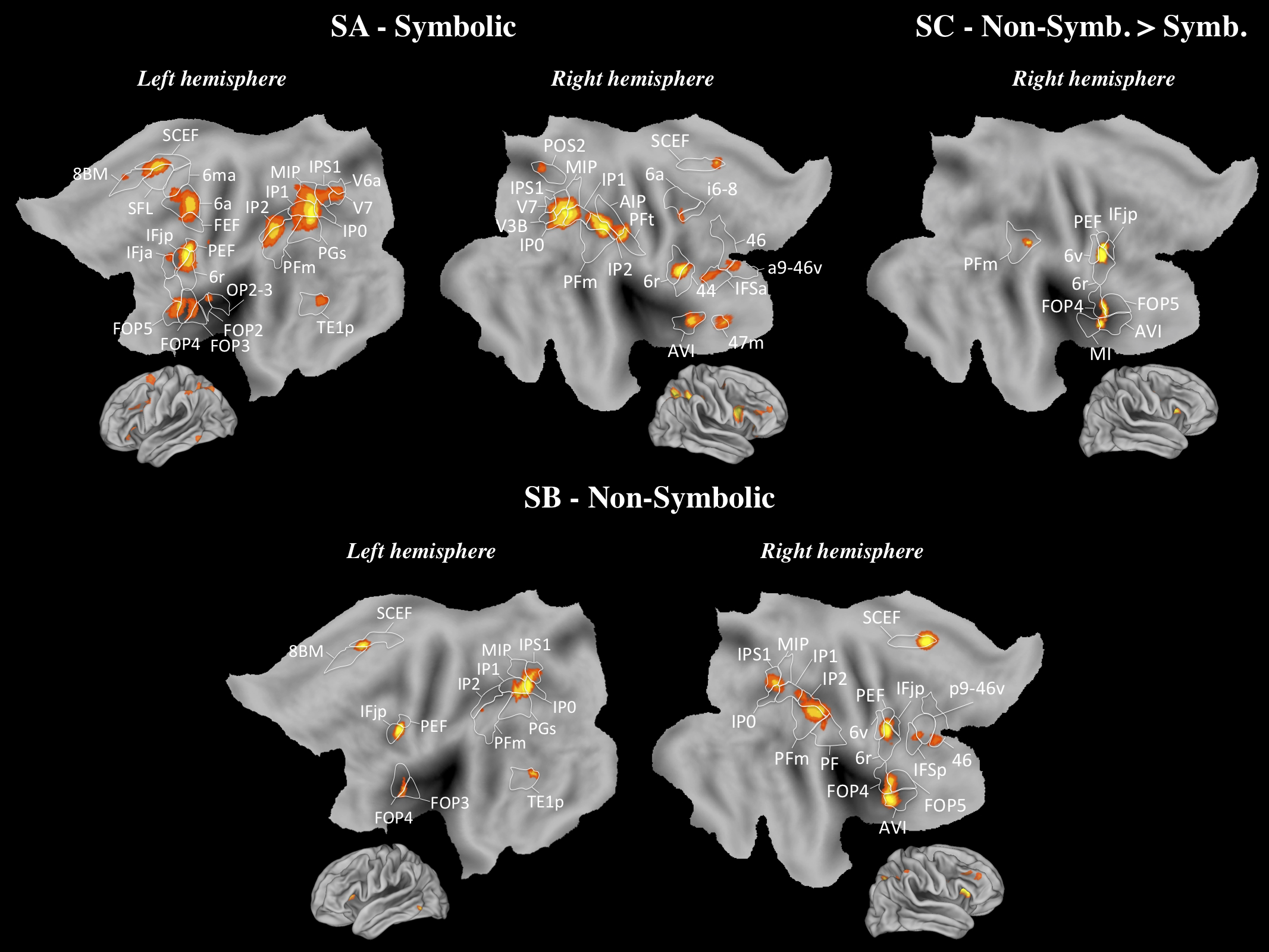
Fig. S1.* *. SYMBOLIC and NON-SYMBOLIC (neuroimaging) with a threshold at 50mm^3^ (same threshold as the one used by Sokolowski et al. (2017). ALE map derived from the studies included in (A) SYMBOLIC and (B) NON-SYMBOLIC, and (C) the NON-SYMBOLIC > SYMBOLIC contrast, viewed on PALS-B12 left and right atlas surface configurations (Van Essen, 2005). Flat maps (Top) and lateral fiducial maps (Bottom). The parcellation is based on Glasser et al. (2016). For abbreviations and explanation, see the main text and Appendix.*

**References**

Sandrini, M., Rossini, P.M., Miniussi, C., 2004. The differential involvement of inferior parietal lobule in number comparison: a rTMS study. Neuropsychologia 42 (14), 1902–1909. doi:10.1016/j.neuropsychologia.2004.05.005.

Sasanguie, D., Göbel, S.M., Reynvoet, B., 2013. Left parietal TMS disturbs priming between symbolic and non-symbolic number representations. Neuropsychologia 51(8), 1528–1533. doi:10.1016/j.neuropsychologia.2013.05.001.

Basso, A., Burgio, F., Caporali, A., 2000. Acalculia, aphasia and spatial disorders in left and right brain-damaged patients. Cortex 36 (2), 265–280. doi:10.1016/S0010-9452(08)70528-8.

Benavides-Varela, S., Pitteri, M., Priftis, K., Passarini, L., Meneghello, F., Semenza, C., 2014. Right-hemisphere (spatial?) acalculia and the influence of neglect. Front. Hum. Neurosci. 8 (644). doi:10.3389/fnhum.2014.00644.

Vogel, S.E., Grabner, R.H., Schneider, M., Siegler, R., Ansari, D., 2013. Overlapping and distinct brain regions involved in estimating the spatial position of numerical and non-numerical magnitudes: an fMRI study. Neuropsychologia 51 (5), 979–989. doi:10.1016/j.neuropsychologia.2013.02.001.

Benavides-Varela, S., Piva, D., Burgio, F., Passarini, L., Rolma, G., Meneghello, F., Smenza, C., 2017. Re-assessing acalculia: distinguish spatial and purely deficits in right-hemisphere damaged patients. Cortex 88, 151–164. doi:10.1016/j.cortex.2016.12.014.

Wei, W., Chen, C., Yang, T., Zhang, H., Zhou, X., 2014. Dissociated neural correlates of quantity processing of quantifiers, numbers, and numerosities. Hum. Brain Mapp. 35 (2), 444–454. doi:10.1002/hbm.22190.

Zago, L., Pesenti, M., Mellet, E., Crivello, F., Mazoyer, B., Tzourio-Mazoyer, N., 2001. Neural correlates of simple and complex mental calculation. Neuroimage 13 (2), 314–327. doi:10.1006/nimg.2000.0697.

Zago, L., Petit, L., Turbelin, M.-.R., Andersson, F., Vigneau, M., Tzourio-Mazoyer, N., 2008. How verbal and spatial manipulation networks contribute to calculation: an fMRI study.

Neuropsychologia 46 (9), 2403–2414. doi:10.1016/j.neuropsychologia.2008.03.001.

Zorzi, M., Priftis, K., Meneghello, F., Marenzi, R., Umiltà, C., 2006. The spatial representation of numerical and non-numerical sequences: evidence from neglect. Neuropsychologia 44(7), 1061–1067. doi:10.1016/j.neuropsychologia.2005.10.025.

Cappelletti, M., Lee, H.L., Freeman, E.D., Price, C., 2009b. The role of right and left parietal lobes in the conceptual processing of numbers. J. Cogn. Neurosci. 22 (2), 331–346. doi:10.1162/jocn.2009.21246.

Cappelletti, M., Muggleton, N., Vincent Walsh, V., 2009a. Quantity without numbers and numbers without quantity in the parietal cortex. Neuroimage 46 (2), 522–529. doi:10.1016/j.neuroimage.2009.02.016.

Chassy, P., Grodd, W., 2012. Comparison of quantities: core and format-dependent regions as revealed by fMRI. Cerebral Cortex 22 (6), 1420–1430. doi:10.1093/cercor/bhr219.

Deloche, G., Souza, L., Willadino-Braga, L., Dellatolas, G., 1999. Assessment of calculation and number processing by adults: cognitive and neuropsychological issues. Percept. Mot. Skills 89 (3), 707–738.

Dormal, V., Andres, M., Dormal, G., Pesenti, M., 2010. Mode-dependent and mode-independent representations of numerosity in the right intraparietal sulcus. Neuroimage 52 (4), 1677–1686. doi:10.1016/j.neuroimage.2010.04.254.

Dormal, V., Andres, M., Pesenti, M., 2008. Dissociation of numerosity and duration processing. Cortex 44 (4), 462–469. doi:10.1016/j.cortex.2007.08.011.

Dormal, V., Andres, M., Pesenti, M., 2012b. Contribution of the right intraparietal sulcus to numerosity and length processing: an fMRI-guided TMS study. Cortex 48 (5), 623–629. doi:10.1016/j.cortex.2011.05.019.

Fias, W., Lammertyn, J., Reynvoet, B., Patrick Dupont, P., Orban, G.A., 2003. Parietal representation of symbolic and nonsymbolic magnitude. J. Cogn. Neurosci. 15 (1), 47–56. doi:10.1162/089892903321107819.

Göbel, S.M., Rushworth, M.F.S., Walsh, V., 2006. Inferior parietal rTMS affects performance in an addition task. Cortex 42 (5), 774–781. doi:10.1016/S0010-9452(08)70416-7.

Menon, V., Rivera, S.M., White, C.D., Glover, G.H., Reiss, A.L., 2000. Dissociating prefrontal and parietal cortex activation during arithmetic processing. Neuroimage 12 (4), 357–365. doi:10.1006/nimg.2000.0613.

Mihulowicz, U., Willmes, K., Karnath, H.-.O., Klein, E., 2014. Single-digit arithmetic processing-anatomical evidence from statistical voxel-based lesion analysis. Front. Hum. Neurosci. 8 (286). doi:10.3389/fnhum.2014.00286.

Piazza, M., Mechelli, A., Price, C.J., Butterworth, B., 2006. Exact and approximate judgements of visual and auditory numerosity: an fMRI study. Brain Res 1106 (1), 177–188. doi:10.1016/j.brainres.2006.05.104.

Pinel, P., Le Clec’H, G., van de Moortele, P.-.F., Naccache, L., Le Bihan, D., Dehaene, S., 1999. Event-related fMRI analysis of the cerebral circuit for number comparison. Neuroreport 10 (7), 1473–1479.

Pinel, P., Piazza, M., Le Bihan, D., 2004. Distributed and overlapping cerebral representations of number, size, and luminance during comparative judgments. Neuron 41 (6), 983–993. doi:10.1016/S0896-6273(04)00107-2.

Priftis, K., Zorzi, M., Meneghello, F., Marenzi, R., Umiltà, C., 2006. Explicit versus implicit processing of representational space in neglect: dissociations in accessing the mental number line. J. Cogn. Neurosci. 18 (4), 680–688. http://dx.doi/org/10.1162/jocn.2006.18.4.680.

Qin, S., Cho, S., Chen, T., Rosenberg-Lee, M., Geary, D.C., Menon, V., 2014. Hippocampal-neocortical functional reorganization underlies children’s cognitive development. Nat Neurosci 17 (9), 1263–1269. doi:10.1038/nn.3788.

Rickard, T.C., Romero, S.G., Basso, G., Wharton, C., Flitman, S., Grafman, J., 2000. The calculating brain: an fMRI study. Neuropsychologia 38 (3), 325–335. doi:10.1016/S0028-3932(99)00068-8.
